# Cardiomyocyte‐Enriched USP20 Ameliorates Pathological Cardiac Hypertrophy by Targeting STAT3 Deubiquitination

**DOI:** 10.1002/advs.202416478

**Published:** 2025-04-07

**Authors:** Lingfeng Zhong, Shanshan Dai, Fan Yu, Guo‐Ping Shi, Qinyan Gong, Yucong Zhang, Jingsi Duan, Zhengyin Lou, Zhixuan Tang, Fuzhe Gong, Derong Chen, Liya Hou, Xinyang Hu, Jinghai Chen, Jian'an Wang, Deling Yin

**Affiliations:** ^1^ Department of Cardiology of the Second Affiliated Hospital Zhejiang University School of Medicine Hangzhou 310009 China; ^2^ State Key Laboratory of Transvascular Implantation Devices Hangzhou 310009 China; ^3^ Heart Regeneration and Repair Key Laboratory of Zhejiang Province Hangzhou 310009 China; ^4^ The Key Laboratory of Emergency and Disaster Medicine of Wenzhou Department of Emergency The First Affiliated Hospital of Wenzhou Medical University Wenzhou 325000 China; ^5^ Department of Medicine Brigham and Women's Hospital Harvard Medical School 77 Avenue Louis Pasteur, NRB‐7 Boston MA 02115 USA; ^6^ Department of Cardiology of First Affiliated Hospital of Wenzhou Medical University Wenzhou 325000 China; ^7^ Institute of Translational Medicine Zhejiang University School of Medicine Hangzhou 310029 China

**Keywords:** cardiac hypertrophy, cardiomyocyte, deubiquitinating enzyme, signal transducer and activator of transcription 3, ubiquitin‐specific protease 20

## Abstract

Although pathological cardiac hypertrophy is a key driver of heart failure, the underlying mechanisms remain incompletely elucidated. This study investigates the role and mechanism of deubiquitinating enzyme (DUB) ubiquitin‐specific protease 20 (USP20) in cardiac hypertrophy. Transcriptomic profiling of hypertrophic hearts shows significant alterations in the expression of DUBs, including a remarkable downregulation of USP20. USP20 is predominantly expressed in cardiomyocytes. Co‐immunoprecipitation (Co‐IP) followed by liquid chromatography‐tandem mass spectrometry (LC‐MS/MS) is used to identify USP20 substrates. Cleavage Under Targets and Tagmentation assay (CUT&Tag) sequencing is employed to identify downstream targets of signal transducer and activator of transcription 3 (STAT3). Functionally, USP20 deficiency exacerbates cardiac hypertrophy induced by either angiotensin II (Ang II) or transverse aortic constriction (TAC), whereas USP20 overexpression alleviates hypertrophic responses. Mechanistically, USP20 deubiquitinates STAT3 by removing K63‐linked ubiquitin chains at K177 via its H645 active site, reducing STAT3 phosphorylation and nuclear translocation. This inhibites STAT3's transcriptional activity at coactivator‐associated arginine methyltransfer (*Carm1)* promoter, leading to upregulated CARM1 expression and mitigated hypertrophy. Importantly, the STAT3 inhibitor Stattic confirms STAT3 serves as a key substrate mediating the cardiac protective effects of USP20. These findings unveil a novel USP20/STAT3/CARM1 axis in cardiomyocytes and reveal its therapeutic potential for cardiac hypertrophy.

## Graphical abstract

Cardiomyocyte‐enriched USP20 regulates the K63‐linked ubiquitination of STAT3, and the cardiomyocyte‐specific USP20‐STAT3‐CARM1 axis exerts a protective role in cardiac hypertrophy. Targeting USP20 through cardiac‐specific gene therapy presents a promising strategy for the treatment of cardiac hypertrophy.

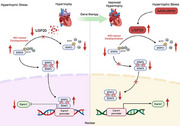

## 1 Introduction

Pathological cardiac hypertrophy is an adaptive response to various forms of cardiovascular stress. Although cardiac hypertrophy can temporarily compensate for cardiac output, it can still lead to adverse cardiac remodeling and, in the long term, heart failure if stress persists.^[^ ^1^, ^2^, ^3^ ^]^ Current therapeutic strategies, such as β‐adrenergic receptor blockers and renin‐angiotensin‐aldosteronesystem inhibitors can improve pathological cardiac hypertrophy, but the high prevalence strongly suggests the involvement of additional regulatory proteins and signaling pathways in pathological cardiac hypertrophy.^[^ ^4^, ^5^, ^6^, ^7^, ^8^ ^]^ Therefore, investigating the pathogenesis of cardiac hypertrophy from diverse perspectives remains a significant challenge and may providing novel therapeutic targets.

Accumulating evidence illustrates that protein ubiquitination plays a crucial role in various cardiac pathologies, including cardiac hypertrophy,^[^ ^9^, ^10^, ^11^, ^12^ ^]^ ischemic heart disease,^[^ ^13^, ^14^ ^]^ and heart failure.^[^ ^15^, ^16^ ^]^ Protein ubiquitination, a complex and dynamic post‐translational modification, is mediated by both E3 ligases and deubiquitinating enzymes (DUBs), which regulate protein function, localization, and degradation.^[^ ^17^ ^]^ Ubiquitin can attach to any of its seven lysine residues (K6, K11, K27, K29, K33, K48, and K63) and form various types of ubiquitin chains, while two types of above are most common (K48‐linked and K63‐linked chains).

K48‐linked ubiquitin chains primarily target proteins for proteasomal degradation, whereas K63‐linked chains are more often involved in non‐degradative processes such as DNA repair and signaling transduction.^[^ ^18^ ^]^ Recent studies have identified two DUBs, ubiquitin‐specific peptidase 25 (USP25) and josephin domain containing 2 (JOSD2), which protect hearts from hypertrophy by regulating the stability of sarcoplasmic/endoplasmic reticulum Ca^2+^‐ATPase 2a (SERCA2a).^[^ ^19^, ^20^ ^]^Additionally, USP28 maintains mitochondrial homeostasis in diabetic cardiomyopathy by modulating the PPARα‐Mfn2 axis.^[^ ^21^ ^]^ Hence, DUBs have emerged as potential therapeutic targets for cardiac hypertrophy and heart failure.

Among the DUB family, ubiquitin‐specific protease 20 (USP20) plays a critical role in regulating protein stability^[^ ^22^ ^]^ and metabolic processes.^[^ ^23^ ^]^ Most studies on USP20 have focused on cancer signaling pathways. For example, a transcription factor Snail homolog 2 (SNAI2), which promotes metastasis, is a highly unstable protein through ubiquitin‐proteasome‐mediated degradation; USP20 stabilizes SNAI2 by deubiquitination, leading to breast cancer metastasis.^[^ ^22^ ^]^ Furthermore, USP20 promotes cancer progression by inhibiting autophagy and enhancing cell proliferation.^[^ ^24^ ^]^ However, the role of USP20 in cardiovascular diseases remains poorly characterized. Recent evidence suggests a protective role for USP20 in atherosclerosis,^[^ ^25^ ^]^ and USP20 positively regulates myocardial β1‐adrenergic receptor expression and signaling in the heart.^[^ ^26^ ^]^ Since USP20 regulates many substrate proteins, the deubiquitination of the downstream regulated by USP20 remains unclear.

Signal transducer and activator of transcription 3 (STAT3), a pivotal member of the STAT family, acts as a transcription factor and plays an essential role in both cardiac physiology and pathophysiology,^[^ ^27^ ^]^ including cell survival, energetics, and metabolism.^[^ ^28^, ^29^ ^]^ Moreover, STAT3 can be stabilized and activated through post‐translational ubiquitination modifications, further amplifying its biological function.^[^ ^30^, ^31^, ^32^ ^]^ In the cardiovascular diseases, the mechanisms by which DUBs modulate STAT3 ubiquitination and subsequently impact its transcriptional regulatory capacity remain poorly understood.

In this study, we identified cardiomyocyte‐enriched USP20 as a protective regulator in human and mouse cardiac hypertrophy. Cardiomyocyte‐specific deficiency of USP20 exacerbated cardiac hypertrophy and dysfunction, whereas cardiomyocyte‐specific overexpression of USP20 ameliorated cardiac function upon hypertrophy. Mechanistically, we identified STAT3 as a substrate of USP20 in cardiomyocytes. USP20 impedes the nuclear transcription factor activity of STAT3 by removing its K63‐linked ubiquitin chains. Our findings reveal a novel cardiomyocyte‐specific USP20‐STAT3‐CARM1 (coactivator‐associated arginine methyltransferase 1) axis that regulates cardiac hypertrophy and heart failure, and position USP20 as a potential therapeutic target for pathological cardiac hypertrophy and heart failure.

## 2 Results

### 2.1 Identification of Cardiomyocyte‐Enriched USP20 As a Downregulated Factor in Mouse and Human Cardiac Hypertrophy

**Figure 1:**
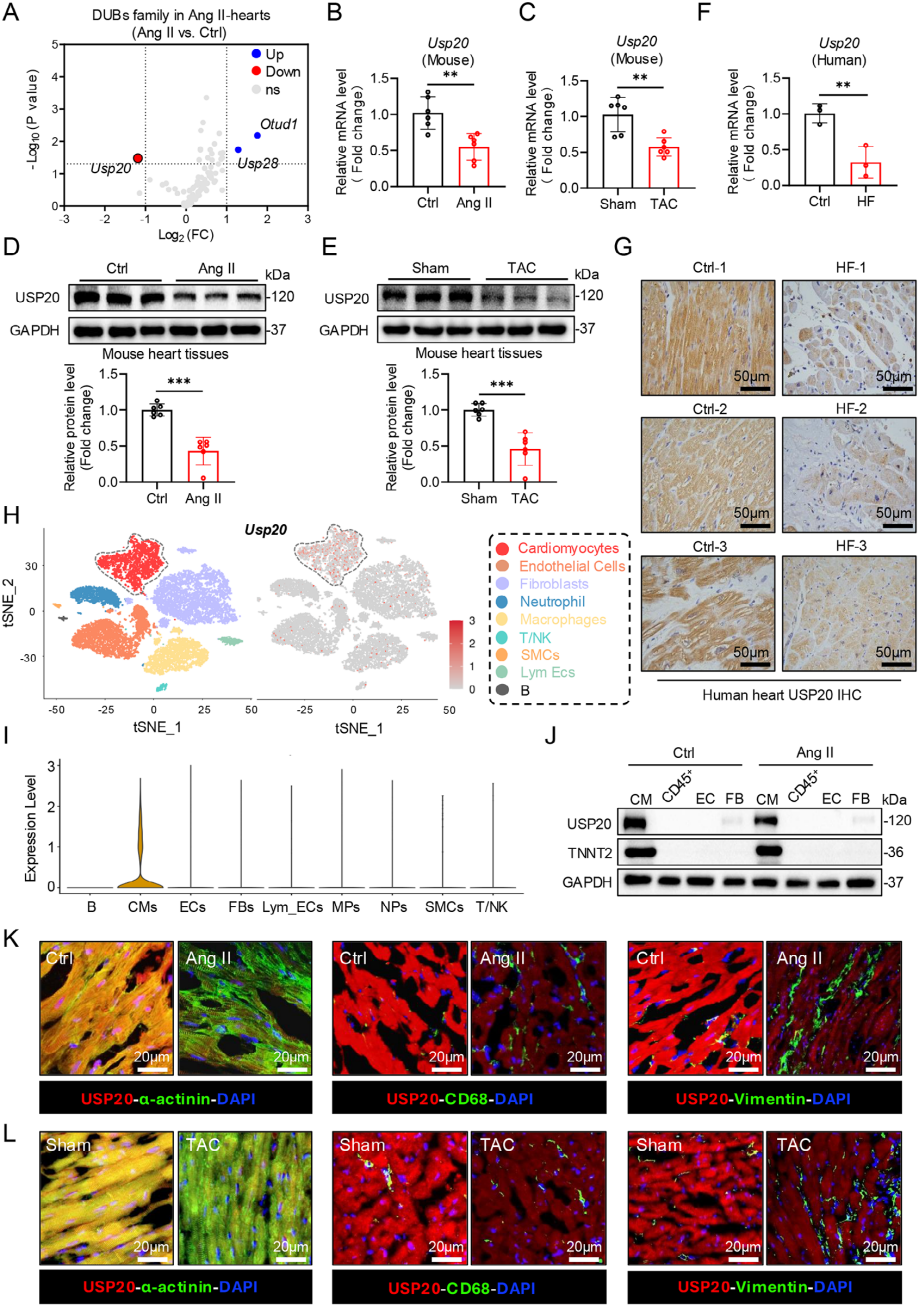
Identification of cardiomyocyte‐enriched USP20 as a downregulated factor in mouse and human cardiac hypertrophy. A) Wild type (WT) male mice were induced hypertrophic myocardium by Ang II (please refer to the Supplementary data online for detailed methods). The heart samples from vehicle and Ang II‐administered mice were harvested for RNA transcriptome sequencing and showed the expression profiles of deubiquitinating enzymes (DUBs). We used log_2_ fold change as the data source of x‐axis and log_10_ P‐value as the data source of y‐axis. Blue dots and red dots represent upregulated and downregulated DUBs compared to the control group, respectively. Gray dots indicate DUBs with no statistically significant difference compared to the control group. B,C) RT‐qPCR analysis of Usp20 mRNA level in Ang II‐ (B) and TAC‐(C) induced hypertrophic myocardium. *n* = 6. **p < 0.01. D,E) The protein level of USP20 in the heart tissues was determined by immunoblotting in Ang II‐ (D) and TAC‐ (E) induced cardiac hypertrophy. *n* = 6. ***p < 0.001. F) RT‐qPCR analysis of Usp20 mRNA level in human hearts from control or patients diagnosed with cardiac hypertrophy. *n* = 3. HF = heart failure. **p < 0.01. G) Representative images of immunohistochemical staining of USP20 as from (F). H,I) Single‐cell mRNA sequencing was conducted on the hearts from Ang II‐administered mice. H) tSNE plot showing 9 main cell types, including Cardiomyocytes (CMs), Endothelial Cells (ECs), fibroblasts (FBs), Neutrophil (NPs), Macrophages (MPs), T/NK cells (T/NK), smooth muscle cells (SMCs) and B cells (B) (Left). Biaxial scatter plot illustrating Usp20 expression patterns across these cell types (Right). I) Violin plot shows that Usp20 expression patterns in these cell types. J) Cardiomyocytes (CMs), fibroblasts (FBs, CD45^−^CD140α^+^), immune cells (CD45^+^), and endothelial cells (CD45^−^CD31^+^) in the heart tissues from vehicle or Ang II‐administrated mice were sorted by flow cytometry and determined USP20 expression. K,L) The cellular origin of USP20 in heart sections in mice induced by Ang II (K) and TAC (L) were assessed using immunofluorescence staining Red: USP20; Green: α‐actinin for cardiomyocyte; CD68 for macrophage; vimentin for fibroblast.

Recent studies suggest that deubiquitinating enzymes (DUBs) contribute to the development of pathological cardiac hypertrophy.^[^ ^33^ ^]^ We first analyzed the mRNA profiles of DUBs families in the hypertrophic myocardium of mice challenged with Angiotensin (Ang II), and found significant alterations in the gene expression of *Otud1*, *Usp28*, and *Usp20* (**Figure** **1**A). As the effects of OTUD1 and USP28 in diseased hearts have been previously documented,^[^ ^21^, ^30^ ^]^ therefore, we sought to investigate the unknown role of USP20 in cardiac pathophysiology. We found that expression level of Usp20 was attenuated in mice hearts induced by either Ang II or transverse aortic constriction (TAC) in both transcriptional level (Figure 1B,C) and protein level (Figure 1D,E), compared to the control or sham group respectively. Importantly, similar results of USP20 were observed in human hypertrophic myocardium of patients with heart failure (Figure 1F,G).

To identify the cell type contributes to the downregulation of USP20 in hypertrophic myocardium, we conducted single‐cell RNA sequencing (scRNA‐Seq) on ≈16 928 single cells from Ang II‐administered mice hearts. Based on specific marker genes expression, we categorized nine cell types: cardiomyocytes (CMs), endothelial cells (ECs), fibroblasts (FBs), neutrophil (NPs), macrophages (MPs), T/NK cells, smooth muscle cells (SMCs), Lymphatic endothelial cells (Lym Ecs) and B cells (Table S1, Supporting Information). Usp20 mRNA expression was predominantly observed in cardiomyocytes (Figure 1H,I). Consistently to scRNA‐Seq data, USP20 showed its absolute predominance expression in cardiomyocytes in immunoblot (Figure 1J). In addition, the protein expression of USP20 in neonatal rat cardiomyocytes (NRCMs) was downregulated upon Ang II treatment in a time‐dependent manner (Figure S1A,B, Supporting Information). Furthermore, immunofluorescence of mouse heart stimulated by either Ang II or TAC showed that the downregulated USP20 was primarily localized in α‐actinin^+^ cardiomyocytes, rather than in CD68^+^ macrophages or vimentin^+^ fibroblasts (Figure 1K,L, Figure S1C,D, Supporting Information). Taken together, we identified that cardiomyocyte‐enriched USP20 expression is downregulated in mouse and human hypertrophic hearts.

### 2.2 Cardiomyocyte‐specific Deficiency of USP20 Exacerbates Cardiac Hypertrophy and Dysfunction Induced by Ang II

**Figure 2:**
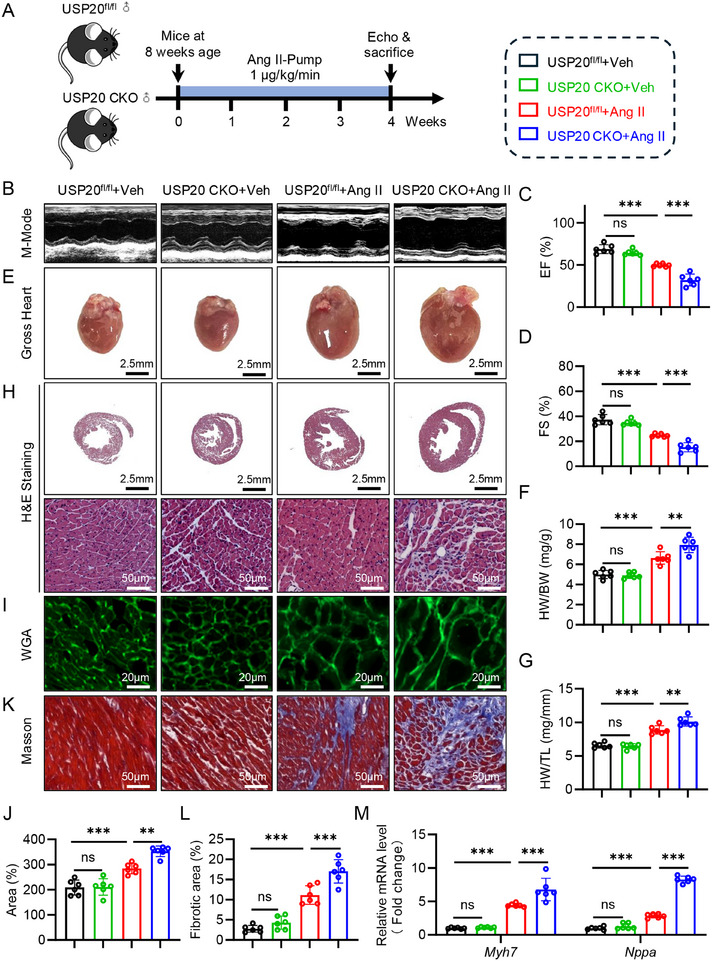
Cardiomyocyte‐specific deficiency of USP20 exacerbates cardiac hypertrophy and dysfunction induced by Ang II. A) Schematic illustration of the experimental design of USP20 in Ang II‐induced cardiac dysfunction and myocardial hypertrophy: USP20^fl/fl^ as control and cardiomyocyte‐specific USP20 deficiency (USP20 CKO) mice were implanted with Ang II‐infused osmotic mini‐pump (1000 ng kg/min) or saline for 4 weeks. B–D) Representative echocardiographic images from each group in mice. *n* = 6 (B). Quantification of ejection fraction (EF) (C) and fractional shortening (FS) (D) of each group, *n* = 6. E) Representative images of gross‐heart of each group. *n* = 6 per group. Scale bar, 2.5 mm. F) The ratio of heart weight (HW) to body weight (BW). G) The ratio of heart weight (HW) to tibial length (TL). H) Representative HE stained images of heart sections. *n* = 6. Scale bar, 2.5 mm and 50 µm. I,J) Representative wheat germ agglutinin (WGA) stained images of heart sections, *n* = 6. Scale bar, 20 µm (I), and quantification of WGA of each group (J). K,L) Representative masson stained images of myocardial interstitium in heart sections. Scale bar, 50 µm (K) and quantitative analysis (L). M) RT‐qPCR analysis of Myh7 and Nppa in the heart tissues, *n* = 6. ns., no significance, **p < 0.01, ***p < 0.001.

We generated cardiomyocyte‐specific USP20 knockout mice (USP20 CKO) by breeding USP20^fl/fl^ mice with *Myh6*‐Cre mice (Figure S2A, Supporting Information) and assessed the expression levels of USP20 across various organs and tissues (Figure S2B,C, Supporting Information). USP20 CKO mice, along with littermate USP20^fl/fl^ mice (as control), were implanted subcutaneously with osmotic mini‐pumps delivering Ang II for 4 weeks (**Figure** **2**A). During Ang II‐induced cardiac hypertrophy, serum Ang II levels did not affect the body weight (BW) of mice (Figure S3A,B, Supporting Information). Both USP20 CKO and USP20^fl/fl^ mice exhibited a significant increase in systolic blood pressure (SBP), and no significant differences between two groups (Figure S3C,D, Supporting Information). Non‐invasive echocardiography results showed that cardiomyocyte deficiency of USP20 caused more pronounced cardiac dysfunction in the mice administrated with Ang II (Figure 2B). This was characterized by decreased ejection fraction and fractional shortening (Figure 2C,D; and Table S4, Supporting Information). USP20 CKO mice showed exacerbated Ang II‐induced heart enlargement (Figure 2E), and increased ratio of heart weight (HW) to BW (Figure 2F), as well as the ratio of HW to tibial length (TL) (Figure 2G). Cardiomyocyte size, as shown by H&E staining (Figure 2H) and wheat germ agglutinin (WGA) staining (Figure 2I,J), exhibited similar patterns of hypertrophy. Furthermore, cardiac fibrosis was significantly increased in Ang II‐administered USP20 CKO mice compared to USP20^fl/fl^ mice assessed with masson's trichrome staining (Figure 2K,L; Figure S3E, Supporting Information). Additionally, the mRNA levels of hypertrophic markers *Myh7* and *Nppa* were significantly increased in the heart tissues of Ang II‐administered USP20 CKO mice compared to USP20^fl/fl^ mice (Figure 2M). And the mRNA levels of fibrosis marker genes *Col‐1a1* and *Tgf‐β* also exhibited a similar trend (Figure S3F, Supporting Information). These findings reveal that cardiomyocyte‐specific knockout of USP20 exacerbates cardiac hypertrophy and dysfunction induced by Ang II.

We then determined the effect of USP20 on cardiomyocyte hypertrophy in vitro. As shown in Supplementary data online Figure S4A–C (Supporting Information), we found that increasing protein level of USP20 further reduced the expression of hypertrophic markers (β‐MYHC, ANP in protein; *Myh7, Nppa* in mRNA). The Ang II‐induced increase of cardiomyocyte surface area was also decreased (Figure S4D,E, Supporting Information). In contrast, decreasing USP20 protein expression using siRNA (si‐USP20) led to opposite effects, with an extra increase in expression of hypertrophic marker and cardiomyocyte surface area (Figure S4F–J, Supporting Information).

### 2.3 Deficiency of Cardiomyocyte‐Specific USP20 Aggravates Cardiac Hypertrophy and Dysfunction Induced by TAC

**Figure 3:**
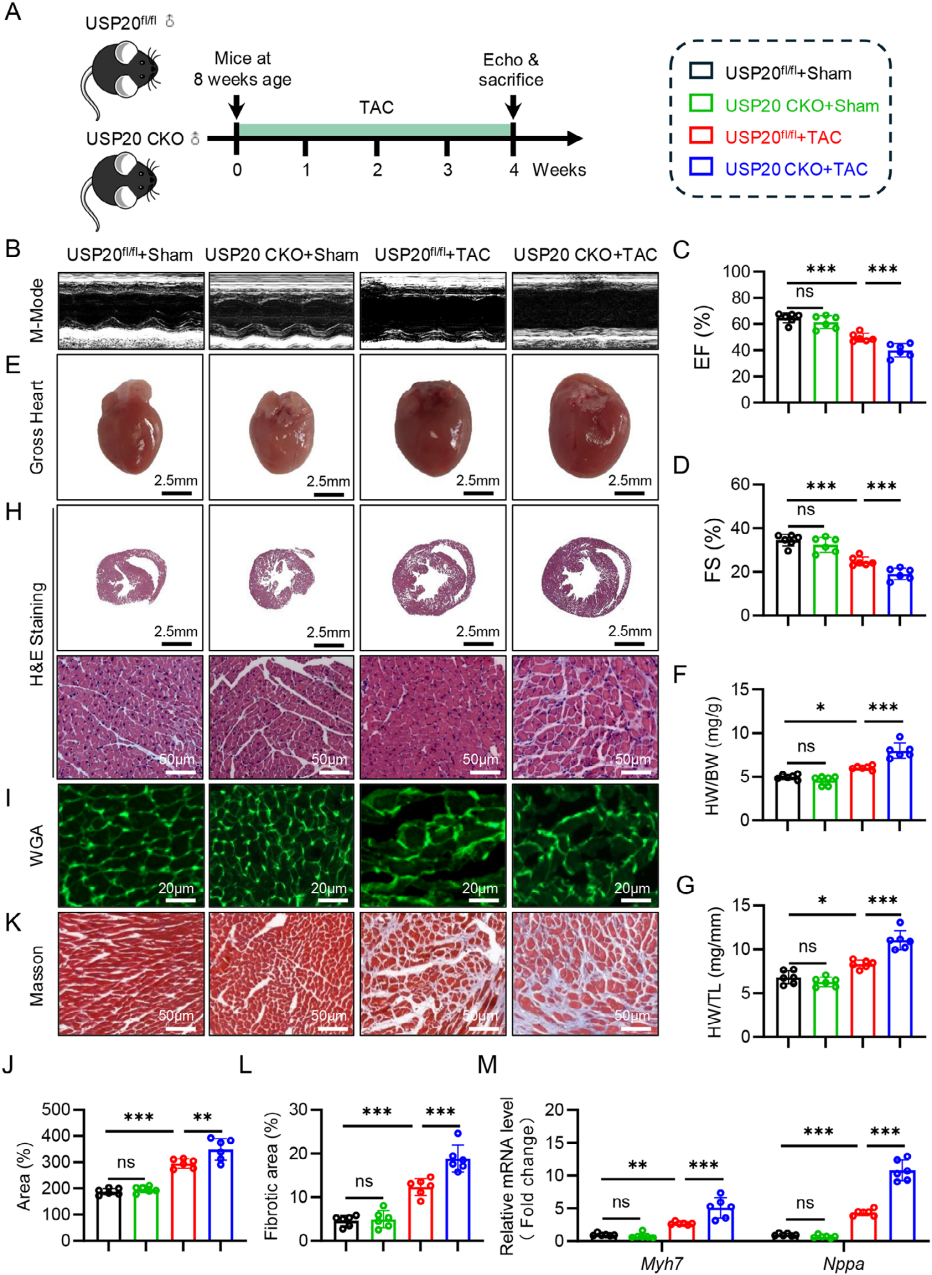
Deficiency of cardiomyocyte‐specific USP20 aggravates cardiac hypertrophy and dysfunction induced by TAC. A) A schematic diagram illustrating the experimental for USP20‐mediated cardiac dysfunction and myocardial hypertrophy induced by TAC: TAC or sham surgery was performed in USP20^fl/fl^ as control and cardiomyocyte‐specific USP20 deficiency (USP20 CKO) mice for 4 weeks. B–D) Representative echocardiographic images from each group in mice (B). *n* = 6. Quantification of ejection fraction (EF) (C) and fractional shortening (FS) (D) of each group *n* = 6. E) Representative images of gross‐heart of each group. *n* = 6. Scale bar, 2.5 mm. F) The ratio of heart weight (HW) to body weight (BW). G) The ratio of heart weight (HW) to tibial length (TL). H) Representative HE stained images of heart sections, *n* = 6. Scale bar, 2.5 mm and 50 µm. I,J) Representative wheat germ agglutinin (WGA) stained images of heart sections, *n* = 6. Scale bar, 20 µm (I), and quantification of WGA of each group (J). K,L) Representative masson stained images of myocardial interstitium in heart sections. Scale bar, 50 µm. (K) and quantitative analysis (L). M) RT‐qPCR analysis of Myh7 and Nppa in heart tissues. *n* = 6. ns., no significance, *p < 0.05, **p < 0.01, and ***p < 0.001.

Next, we investigated whether USP20 played an important role in transverse aortic constriction (TAC)‐induced cardiac hypertrophy in both USP20 CKO mice and USP20^fl/fl^ mice (**Figure** **3**A). Post‐procedure, we assessed the aortic blood flow velocity at the construction site using flow doppler ultrasound, observing a significant increase in blood flow velocity following TAC compared to the sham group (Figure S5A,B, Supporting Information). Echocardiography showed that TAC‐induced USP20 CKO mice exhibited more severe cardiac dysfunction than TAC‐induced USP20^fl/fl^ control mice (Figure 3B–D; and Table S5, Supporting Information). Moreover, USP20 CKO mice appeared exacerbated cardiac hypertrophic phenotype in response to TAC, including gross heart size (Figure 3E), HW/BW (Figure 3F), and HW/TL (Figure 3G). Pathological examination further confirmed the aggravation of myocardial hypertrophy and fibrosis resulted from deficiency of USP20. (Figure 3H–L; Figure S5C, Supporting Information). USP20 CKO also resulted in higher mRNA levels of *Myh7*, *Nppa*, *Col‐1a1* and *Tgf‐β* after TAC (Figure 3M; Figure S5D, Supporting Information). These findings (Figures 2 and 3) clarify that USP20 exerts a protective role in both TAC‐induced and Ang II‐induced cardiac hypertrophy in cardiomyocytes, suggesting USP20 a potential therapeutic target for preventing pathological cardiac hypertrophy.

### 2.4 Identification of STAT3 As a Substrate of USP20 in Cardiomyocytes

**Figure 4:**
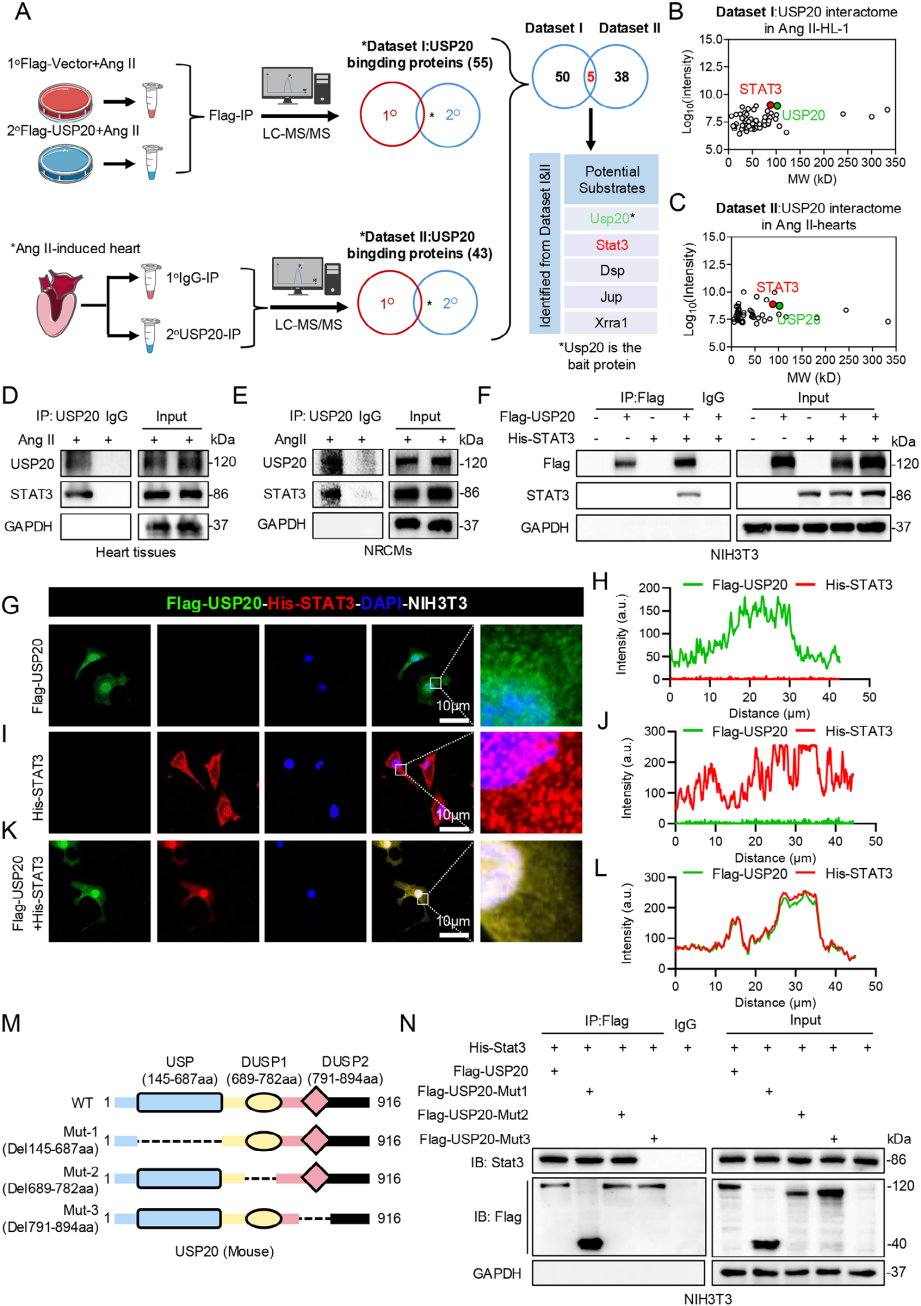
Identification of STAT3 as a substrate of USP20 in cardiomyocytes. A) Schematic illustration of the experimental design for the two interactomes used for USP20 substrate screening. HL‐1 cells (Dataset I) were transfected with either Flag‐vector or Flag‐USP20 following Ang II stimulation at 1 µmol for 24 h. The heart tissues (Dataset II) from mice induced by Ang II were lysed and divided into two portions. The binding proteins were extracted and digested into peptides, and analyzed by liquid chromatography‐mass spectrometry/mass spectrometry (LC‐MS/MS). The list is the potential USP20 substrates identified from the interactomes. B,C) 2D plots with the log_10_ signal intensity of the quantified proteins on the y axis and the molecular weight (MW) of proteins on the x axis were identified from Dataset I (B) and II (C). D) Immunoprecipitation (IP) of USP20 and STAT3 in the heart tissues from Ang II‐ or vehicle‐treated mice. E) IP of USP20 and STAT3 in Ang II‐incubated neonatal rat cardiomyocytes (NRCMs). F) Co‐IP assays were conducted using NIH3T3 cells transfected with Flag‐USP20 or His‐STAT3, as well as cells co‐transfected with Flag‐USP20 and His‐STAT3 plasmids. G,H) Immunofluorescence of exogenous Flag‐USP20 (green) in NIH3T3 transfected Flag‐USP20 plasmid (G) and quantitative analysis (H). The immunofluorescence of His (red) was performed to exclude non‐specific signals from His antibody and the TRITC‐labeled secondary antibody. I,J) Immunofluorescence of exogenous His‐STAT3 (red) in NIH3T3 transfected His‐STAT3 plasmid (I) and quantitative analysis (J). The immunofluorescence of Flag (green) was performed to exclude non‐specific signals from Flag antibody and the Alexa Fluor 488‐labeled secondary antibody. K,L) Co‐localization of exogenous Flag‐USP20 (green) and His‐STAT3 (red) in NIH3T3 expressing Flag‐USP20 and His‐STAT3 (K) and quantitative analysis (L). M) Schematic illustration of the USP20 domain deletion construct used in (N). N) Co‐IP of wt‐USP20, mut‐USP20, and STAT3 in NIH3T3 cells co‐transfected with overexpression plasmids of Flag‐wt‐USP20, Flag‐mut‐USP20 and His‐STAT3. Exogenous normal or mutated USP20 was immunoprecipitated with anti‐Flag antibody.

DUBs exert biological activities by degrading proteins or modulating function of substrates.^[^ ^17^ ^]^ To delineate the candidate substrates of USP20 in cardiac hypertrophy, we utilized myocardial tissues from Ang II‐induced mice in vivo and mouse‐derived HL‐1 cells in vitro respectively to perform co‐immunoprecipitation (Co‐IP) coupled with liquid chromatography‐mass spectrometry/mass spectrometry (LC‐MS/MS) to screen potential USP20 substrates, the workflow for this interactome analysis is illustrated in **Figure** **4**A. Excluding peptides related to antibody light and heavy chains and selecting target proteins with a fold change greater than 2.0, we identified that 55 USP20 physically binding proteins in HL‐1 cells (dataset I, Figure 4B), and 43 binding proteins in heart tissues (dataset II, Figure 4C). By overlapping these two interactomes, we pinpointed potential substrates of USP20: STAT3, DSP, JUP, XRRA1. Interestingly, KEGG enrichment analysis indicated that the JAK‐STAT pathway is significantly involved in USP20‐mediated cardiac hypertrophy (Figure S6A, Supporting Information). Therefore, our results suggest that USP20 may prevent cardiac hypertrophy through STAT3 as a substrate.

We next performed validation of the mass spectrometry by immunoprecipitation and found that USP20 interacts with STAT3 in heart tissues and neonatal rat cardiomyocytes (NRCMs) following Ang II treatment (Figure 4D,E), suggesting that USP20 directly binds to the STAT3 protein in cardiomyocytes. Next, we verified the binding between USP20 and STAT3 by co‐transfection of Flag‐USP20 and His‐STAT3 plasmids into NIH3T3 cells, with no significant binding detected in the cells transfected with Flag‐USP20 or His‐STAT3 and IgG control group. (Figure 4F). Additionally, immunofluorescence analysis confirmed the co‐localization of both exogenous and endogenous USP20 and STAT3 in NIH3T3 cells (Figure 4G–L) and NRCMs (Figure S6B,C, Supporting Information). USP20 is composed of four distinct structural domains: the N‐terminal zinc‐finger ubiquitin‐binding domain (ZnF‐UBP), the catalytic (USP) domain, and two tandem DUSP domains (DUSP1, DUSP2).^[^ ^34^ ^]^ The ZnF‐UBP domain of USP20 may play a physiological role unrelated to its ubiquitin‐binding capacity.^[^ ^34^ ^]^ We generated three USP20 mutants to delineate which domain interacts with STAT3 (Figure 4M). By co‐transfecting STAT3 and the mutated USP20 plasmids into NIH3T3 cells, we found that USP20 could not bind to STAT3 when amino acids 791 to 894 were deleted, whereas mutations in other domains did not affect such binding (Figure 4N). Taken together, we identified STAT3 is a substrate of USP20 in cardiomyocytes, in which USP20 directly binds to STAT3 through its DUSP2 domain.

### 2.5 USP20 Attenuates the K63‐Linked Deubiquitination of STAT3 at Residue K177

**Figure 5:**
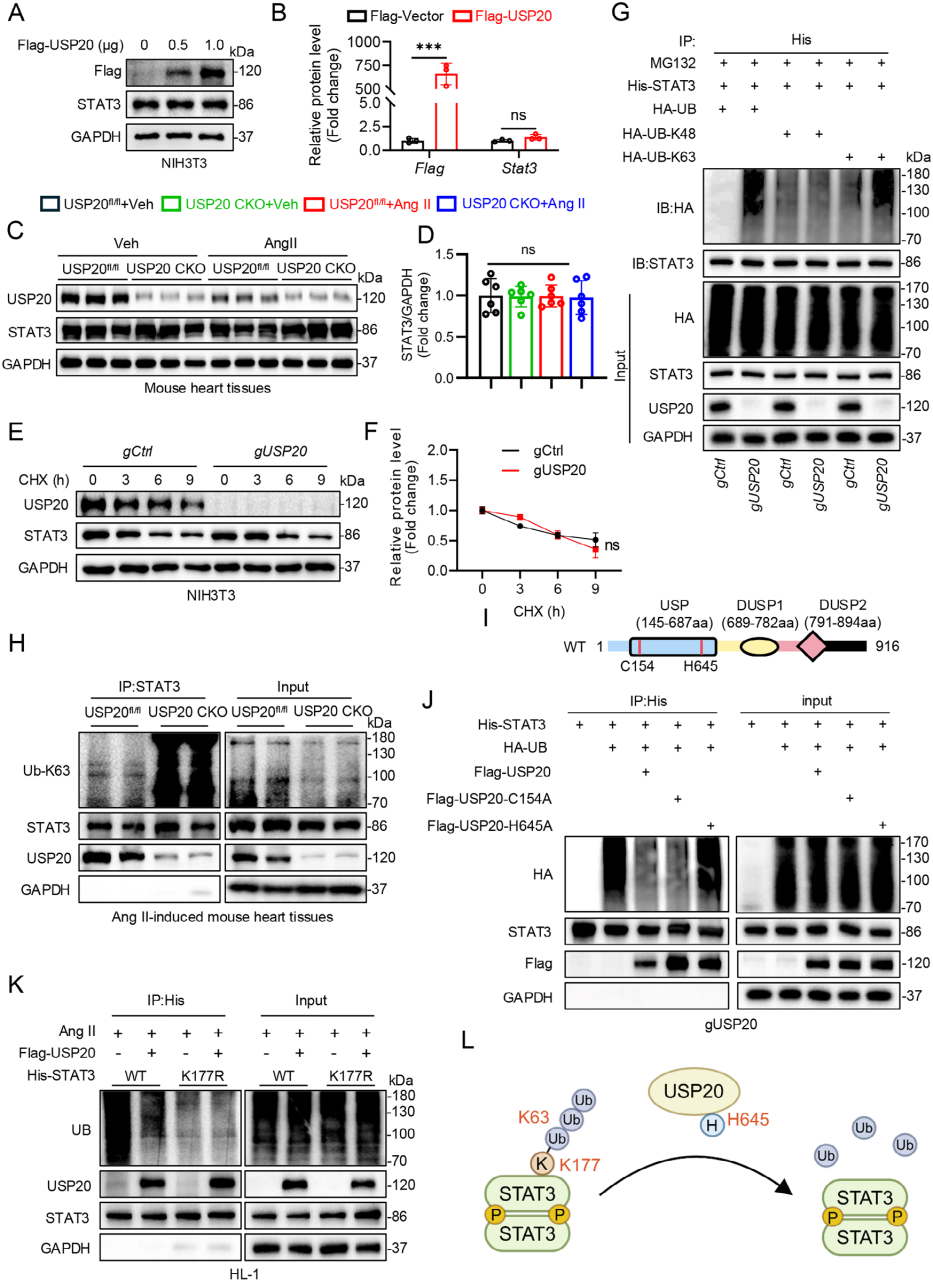
USP20 attenuates the K63‐linked deubiquitination of STAT3 at residue K177 through the active site H645. A,B) Overexpression of USP20 Flag‐plasmid was transfected into NIH3T3 cells for 24 h. The levels of Flag and STAT3 were assesses by immunoblotting (A) and the corresponding quantitative analysis (B) *n* = 3. n.s., no significance, ***p < 0.001. C,D) USP20 CKO and USP20^fl/fl^ mice were induced myocardial hypertrophy by Ang II. USP20 and STAT3 expression (C) in the hearts was determined as in (A) and the quantitative analysis of STAT3 (D). *n* = 6. ***p < 0.001, ns., no significance. E,F) NIH3T3 cells were infected with lentivirus containing empty vector (gCtrl) or USP20‐gRNA (gUSP20) at multiplicith of infection (MOI) of 50. After antibiotic selection, USP20^−/−^‐NIH3T3 cells were obtained. Either gCtrl or gUSP20 were incubated with cycloheximide (CHX). The USP20 and STAT3 expression (E) was examined as in (A) and densitometric quantification of STAT3 (F). *n* = 3. ns., no significance. G) NIH3T3 cells were co‐transfected either gCtrl or gUSP20 with overexpression plasmids of His‐STAT3, HA‐Ub, HA‐K48, and HA‐K63, and then incubated with MG132 at 10 µmol for IP of STAT3. Ubiquitinated STAT3 was detected by immunoblotting with an His‐specific antibody to clarify the ubiquitination pattern of STAT3 regulated by USP20. H) IP of STAT3 in the heart tissues of USP20^fl/fl^ or USP20 CKO mice treated with Ang II for induction of myocardial hypertrophy. Ubiquitinated STAT3 was determined by immunoblotting using an Ub‐K63 antibody to clarify the K63 ubiquitination level of STAT3 regulated by USP20. I) Schematic illustration of the USP20 active site deletion construct used in J. J) IP of STAT3 in gUSP20 that co‐transfected with overexpression plasmids of His‐STAT3, HA‐Ub, Flag‐USP20, Flag‐USP20^C154A^ and Flag‐USP20^H645A^. Exogenous ubiquitinated STAT3 was detected by immunoblotting using an His‐specific antibody to identify the active site of USP20 regulating ubiquitination of STAT3. K) IP of STAT3 in Ang II‐incubated HL‐1 cells that co‐transfected with overexpression plasmids of His‐STAT3^WT^, His‐STAT3^K177R^, Flag‐USP20. Exogenous ubiquitinated STAT3 was detected by immunoblotting using an His‐specific antibody to identify the ubiquitination site of STAT3 regulated by USP20. L) Schematic illustration showing that USP20 attenuates the K63‐linked deubiquitination of STAT3 at residue K177 through the active site H645.

Previous studies have demonstrated that STAT3 can be modified by K63‐linked ubiquitin chains, which in turn affects its phosphorylation and protein function.^[^ ^35^ ^]^ This post‐translational modification plays a crucial role in regulating the activity of STAT3 in various cellular processes, including its stability, subcellular localization, and interaction with other proteins.^[^ ^36^ ^]^ Given that USP20 is a deubiquitinase that directly binds to STAT3, we determined whether USP20 could modulate the K63 ubiquitination of STAT3, thereby influencing its biological activity. Overexpression of Flag‐USP20 did not increase the STAT3 protein level in NIH3T3 cells (**Figure** **5**A,B). The same phenomenon was observed in Veh‐ or Ang II‐treated USP20^fl/fl^ and USP20 CKO mice (Figure 5C,D). We then generated USP20‐knockout NIH3T3 cells (*gUSP20*) by using CRISPR/Cas9 technology. When cycloheximide (CHX) was added to USP20‐knockout cells to inhibit new protein synthesis, the degradation rate of STAT3 protein remained unchanged (Figure 5E,F). Ubiquitin has seven Lys residues (K6, K11, K27, K29, K33, K48, and K63) that can form polyubiquitin chains, in which K48 and K63 linkages are the most prevalent.^[^ ^18^ ^]^ Thus, we transfected *gUSP20* cells with mutated ubiquitin plasmids containing only active K48 (HA‐K48) or K63 (HA‐K63) sites and observed that the ubiquitin molecules on STAT3 were significantly increased in *gUSP20* cells transfected with the HA‐K63 plasmid compared to wild‐type cells (gCtrl) (Figure 5G). Moreover, the K63 regulated ubiquitination level of STAT3 protein was much higher in myocardium tissues of Ang II‐induced USP20 CKO mice than those of USP20^fl/fl^ mice (Figure 5H). Together, these results demonstrate that USP20 removes K63‐linked ubiquitin molecules from STAT3.

DUBs can cleave the amide bonds between ubiquitin molecules and substrate using active sites such as cysteine and histidine.^[^ ^37^ ^]^ To explore the specific roles of these active sites in USP20, we generated mutants by substituting glycine for cysteine at position 154 (USP20^C154A^) and histidine at position 645 (USP20^H645A^) (Figure 5I), both of which are highly conserved across species (Figure S7A, Supporting Information). Then the mutants, USP20^C154A^ and USP20^H645A^, were evaluated for their ability to bind and deubiquitinate STAT3. Our results showed that both mutants could still bind to STAT3 (Figure S7B, Supporting Information). However, the USP20^C154A^ mutant retained its deubiquitination activity, while the USP20^H645A^ could no longer remove ubiquitn molecules from STAT3 (Figure 5J). To further elucidate the role of the H645 site in cardiomyocytes, we assessed the impact of the H645 mutation on USP20's protective effects against Ang II‐induced cardiomyocyte hypertrophy in vitro. The mutation of H645 abolished USP20's protective effect against Ang II‐induced cardiomyocyte hypertrophy. The H645 mutation abolished the protective effect of USP20 on Ang II‐induced cardiomyocyte hypertrophy. Specifically, in NRCMs overexpressing USP20 (H645A), Ang II treatment resulted in significantly higher protein (Figure S7C,D, Supporting Information) and mRNA levels (Figure S7E, Supporting Information) of hypertrophic markers Myh7 and Nppa, as well as increased cardiomyocyte size (Figure S7F,G, Supporting Information), compared to the USP20 (WT) overexpression group. However, these levels were similar to those observed in the null treatment group. These findings reveal that USP20 promotes STAT3 deubiquitination and function through the active site H645, and this regulation is crucial for its protective role against cardiac hypertrophy.

STAT3 consists of six domains: N Domain (ND), Coil‐coil Domain (CCD), DNA Binding Domain (DBD), Linker Domain (LD), SH2 Domain (SH_2_D), and TAD Domain (TAD).^[^ ^38^ ^]^ We created six STAT3 truncation mutants, each lacking one of these domains (Figure S8A, Supporting Information), to determine which domain is responsible for binding USP20. After co‐transfection of these mutants with Flag‐USP20 plasmid in HEK293T cells, result showed that the absence of amino acids 130–320, which constitute the CCD domain, disrupted the binding between STAT3 and USP20 (Figure S8B, Supporting Information). These results suggest that STAT3 binds to USP20 through its CCD domain, which is known to regulate the phosphorylation and nuclear translocation of STAT3 via allosteric regulation of SH2.^[^ ^39^ ^]^ As for the ubiquitination sites on STAT3 involved in pathological cardiac hypertrophy have not been reported, we utilized affinity‐based ubiquitin peptide enrichment ubiquitomics to investigate the specific ubiquitination sites on STAT3 modulated by USP20 (Figure S9A, Supporting Information). Through analyzed the results, we identified 15 potential ubiquitination lysine residues K87, K97, K161, K177, K244, K294, K348, K354, K548, K551, K601, K615, K626, K631 and K707 in STAT3 (Figure S9B, Supporting Information). Given that USP20 binds to the CCD domain (130‐320aa) of STAT3, we focused on lysine within this region (K161, K177, K244, and K294) and constructed mutant plasmids with lysine‐to‐arginine substitutions to simulate the deubiquitinated state of STAT3. Interestingly, the expression of p‐STAT3 in nuclear is significantly decreased when K177, but not the other lysine residues, was mutated (Figure S9C,D, Supporting Information). We then focused on K177 and examined its role in the USP20‐mediated STAT3 ubiquitination in Ang II‐incubated cardiomyocytes. We observed that the K177R mutation did not disrupt USP20‐STAT3 binding but resulted in significantly lower STAT3 ubiquitination compared to the wild‐type, and this reduction was not further decreased by USP20 overexpression (Figure 5K). Taken together, USP20 eliminates the K63‐linked deubiquitination of STAT3 at residue K177 through active site H645 of USP20 (Figure 5L).

### 2.6 USP20 Protects Heart from Cardiac Hypertrophy by Inhibiting STAT3 Nuclear Transcription to Promote CARM1 Expression

**Figure 6:**
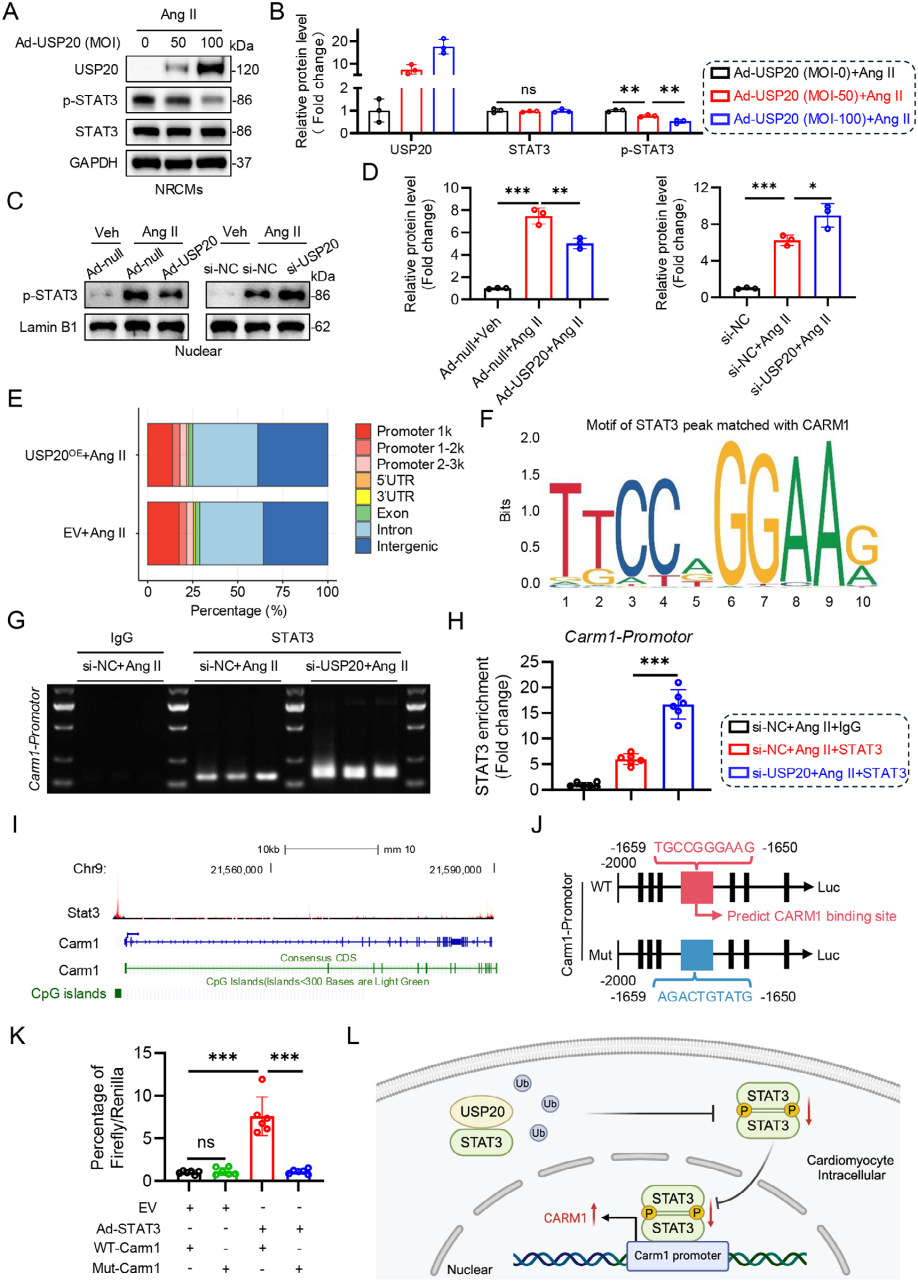
USP20 protects heart from cardiac hypertrophy by inhibiting STAT3 nuclear transcription to promote CARM1 expression. A,B) NRCMs were infected with adenovirus (Ad) encoding USP20 (Ad‐USP20) of multiplicity of infection at MOI of 50 or 100 and Ad‐null as control, then incubated with Ang II at 1 µmol for 24 h. The expression of USP20, p‐STAT3 and STAT3 was examined (A) and quantitative analysis (B). *n* = 3. **p < 0.01, ns., no significance. C,D) NRCMs were transfected with Ad‐Null or Ad‐USP20, and si‐NC (NC, negative control) or si‐USP20 following stimulation with Ang II at 1 µmol for 24 h. Western blotting for p‐STAT3 and Lamin B1 in nuclear (C) and the quantitative analysis (D). *n* = 3. *p < 0.05, **p < 0.01, and ***p < 0.001. E) HL‐1 cells were transfected with plasmids of EV (empty vector) or USP20^OE^ and stimulated with Ang II for cleavage under targets and tagmentation (CUT&Tag) assay. F) The motif of STAT3 peak matched with CARM1. G) CUT&Tag was performed on si‐NC or si‐USP20‐transfected HL‐1 cells to verify STAT3 binding to the promoter regions of the Carm1 gene. H) CUT&Tag‐qPCR assay for the binding of STAT3 at Carm1 promoter regions. *n* = 6. ***p < 0.001. I) The analysis of Chip‐seq data of STAT3 in mouse available in the ENCODE database predicted that STAT3 binds to the promoter region of Carm1. J) Schematic diagram of the construction of wild type (WT) and mutant (Mut) luciferase reporter plasmids of Carm1 promoter. K) NIH3T3 cells were transfected with WT‐Carm1 or Mut‐Carm1 luciferase reporter plasmid and either WT‐STAT3 vector or control vector. The transfected cells were analyzed for luciferase activity. *n* = 6. ***p < 0.001. L) The schematic illustrates that USP20 inhibits nuclear translocation and promoter regions (e.g., Carm1) binding of STAT3, thereby regulating gene expression through STAT3 in cardiomyocytes.

STAT3 serves multiple biological functions, primarily acting as a transcription factor that regulates the expression of numerous genes coding for various proteins.^[^ ^31^ ^]^ Therefore, we further evaluated whether USP20 could enhance the nuclear translocation of STAT3. Previous study reported that STAT3 activation is controlled by phosphorylation at tyrosine 705 (pTyr705).^[^ ^40^, ^41^ ^]^ Therefore, we determined the level of p‐STAT3 at Try705 following USP20 overexpression in Ang II‐stimulated NRCMs. Overexpression of USP20 remarkably decreased the level of p‐STAT3 without affecting the total STAT3 expression (**Figure** **6**A,B). Notably, USP20 overexpression reduced the nuclear translocation of p‐STAT3 in Ang II‐incubated NRCMs, whereas silencing USP20 significantly increased p‐STAT3 level in nuclear (Figure 6C,D). Stattic is a cell‐permeable inhibitor that specifically targets the STAT3 protein, preventing its activation and dimerization.^[^ ^42^ ^]^ By blocking the SH2 domain, stattic prevents STAT3 from translocating to the nucleus and initiating the transcription of target genes. Interestingly, inhibition STAT3 by STAT3 inhibitor stattic attenuates STAT3 phosphorylation and nuclear translocation as well (Figure S10A,B, Supporting Information). Karyopherin alpha 3 (KPNA3) has been identified as an auxiliary protein to facilitate STAT3 nuclear translocation.^[^ ^43^ ^]^ We then determined whether USP20 binding to STAT3 could interfere with the STAT3‐KPNA3 association, thereby reducing nuclear translocation of STAT3. IP assay showed that Ang II stimulation enhanced the interaction between STAT3 and KPNA3, however, this interaction was diminished when USP20 was overexpressed in NRCMs (Figure S10C, Supporting Information).

To identify the potential downstream targets of STAT3 following Ang II stimuli, we performed cleavage under targets and tagmentation (CUT&Tag) assay with high‐throughput sequencing. The results showed that STAT3 peak‐related genes significantly decreased within 1000 bp (Promotor 1k) of the gene transcriptional start site (TSS) (Figure 6E, from 17.45% to 13.86%). Subsequently, we analyzed the up‐regulated genes in *EV+Ang II* versus *USP20^OE^+ Ang II* conduction and identified significant differences in the expression of cardiac function‐related genes such as *Stk3*, *Pten*, *Eno1*, *Atf4*, *Alkbh5*, coactivator‐associated arginine methyltransferase 1 *(Carm1)* and *Pcna* (Figure S10D, Supporting Information). RT‐qPCR analysis showed that, apart from increased expression of *Carm1* and *Atf4*, the expression levels of other genes did not show significant alterations in USP20^OE^‐Ang II‐incubated NRCMs (Figure S10E, Supporting Information). CARM1 is a crucial factor for cardiac homeostasis and regulates multiple aspects of cardiomyocyte maturation, including cellular hypertrophic growth and myofibril expansion.^[^ ^44^ ^]^ Given the unclear role of CARM1 in pathological cardiac hypertrophy, we employed si‐CARM1 to downregulate CARM1 expression in NRCMs. Upon Ang II stimulation, we observed that si‐CARM1 led to a more pronounced hypertrophic response, characterized by upregulation of hypertrophic marker genes at both the protein (Figure S10F,G, Supporting Information) and mRNA levels (Figure S10H, Supporting Information), as well as an increase in cardiomyocyte surface area (Figure S10I,J, Supporting Information). We hypothesized that USP20‐STAT3 axis ameliorates cardiac hypertrophy through transcriptional regulation of *Carm1*. To determine the binding motif between STAT3 and *Carm1*, we predicted the binding sites of STAT3 to the *Carm1* promoter regions by JASPAR (Figure 6F). We analyzed the potential binding site which clustered in the ‐1659–1650 bp of the *Carm1* promoter regions. Using CUT&Tag and qPCR assays, we found that STAT3 directly bound to the promoter regions of *Carm1*, which was significantly increased by USP20 silencing (Figure 6G,H, Supporting Information). On the other hand, this binding was decreased when USP20 was overexpressed (Figure S10K,L, Supporting Information). We also predicted that STAT3 peak enriched to the promoter region of *Carm1* by using ENCODE database (Figure 6I). Luciferase assays were performed by cloning the mutated ‐1659 to ‐1650 bp fragments of the *Carm1* promoter (Mut‐Carm1), which lacked the putative STAT3‐binding site, and using the wild‐type fragment as a control (Figure 6J). Transfection of STAT3‐overexpressing in NIH3T3 cells with either wild‐type or mutant *Carm1* promoter plasmids showed increased luciferase expression in vectors containing WT‐Carm1, but not in those with Mut‐Carm1 (Figure 6K). Taken together, these results suggest that USP20 inhibits STAT3 transcriptional activity in nuclear, leading to promoting *Carm1* expression in cardiomyocytes (Figure 6L).

### 2.7 Cardiomyocyte‐Specific Overexpression of USP20 Improves Cardiac Hypertrophy and Dysfunction Induced by Ang II

**Figure 7:**
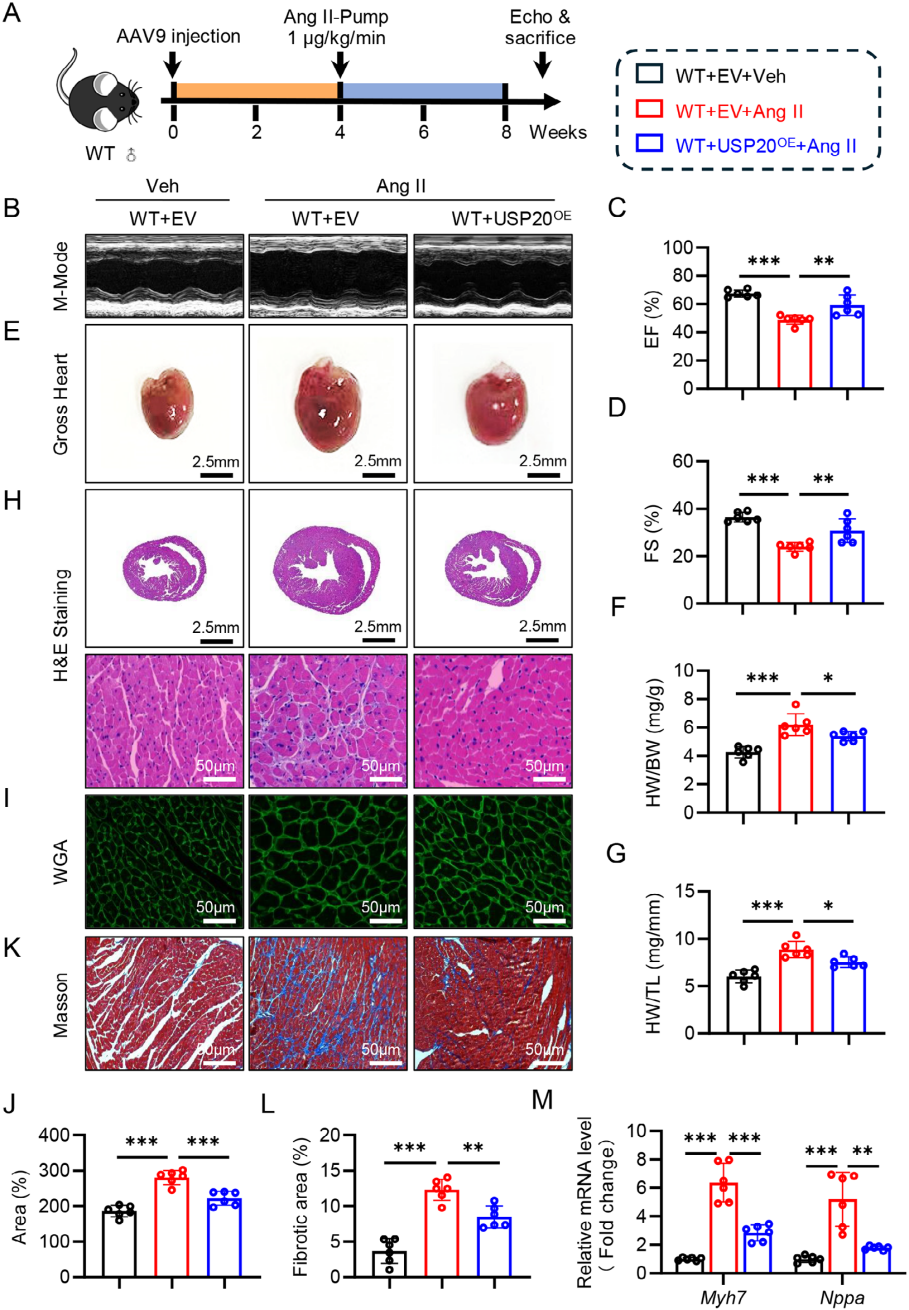
Cardiomyocyte‐specific overexpression of USP20 improves cardiac hypertrophy and dysfunction induced by Ang II. A) WT mice were injected with adeno‐associated virus serotype 9 (AAV9) mediated cardiomyocyte‐specific overexpression of USP20 (USP20^OE^) or empty vector (EV) (2 × 10^11^ v.g., i.v.). Four weeks later, the mice were induced to cardiac hypertrophy by Ang II as in Figure 2A. B) Echocardiographic images from each group in mice. C,D) Echocardiographic analysis of ejection fraction (EF) and fractional shortening (FS). E) Gross‐heart from each group. F) The ratio of heart weight (HW) to body weight (BW). G) The ratio of heart weight (HW) to tibial length (TL). H) HE stained images of heart sections. Scale bar, 2.5 mm and 50 µm. I,J) Representative wheat germ agglutinin (WGA) stained images of heart sections. Scale bar, 50 µm (I) and quantitative analysis (J). K,L) Representative masson stained images of myocardial interstitium in heart sections. Scale bar, 50 µm (K) and quantitative analysis (L). M) RT‐qPCR analysis of Myh7 and Nppa in heart tissues. *n* = 6. ns., no significance, *p < 0.05, ** *p* < 0.01, and ***p < 0.001.

We next evaluated whether USP20 overexpression has a therapeutic effect on myocardial hypertrophy. We prepared a cardiomyocyte‐targeting adeno‐associated virus serotype 9 (AAV9) vector and the wide type mice were subjected to AAV9 carrying USP20 (USP20^OE^) or empty vehicle (EV) respectively via tail vein injection. After 4 weeks, the mice were administered with Ang II to induce hypertrophy and cardiac dysfunction (**Figure** **7**A) and verified their effectiveness (Figure S11A, Supporting Information). AAV9 administration did not alter both body weight (Figure S11B, Supporting Information) and blood pressure in Ang II‐challenged mice (Figure S11C,D, Supporting Information). USP20 overexpression strikingly alleviated cardiac dysfunction induced by Ang II (Figure 7B–G; and Table S6, Supporting Information), and also resulted in reductions in cardiomyocyte size (Figure 7H–J), fibrosis (Figure 7K,L; Figure S11E,F, Supporting Information), and hypertrophic markers (Figure 7M). Alternatively, overexpression of USP20 significantly reduced heart failure‐related serum biomarkers, such as serum ANP (Figure S11G, Supporting Information) and serum BNP (Figure S11H, Supporting Information). Thus, USP20 overexpression mitigates Ang II‐induced cardiac remodeling and heart failure. More importantly, USP20 overexpression did not change the protein level of STAT3 (Figure S11I, Supporting Information), and as expected, USP20 overexpression decreased the K63‐ubiquitination of STAT3 in heart tissues (Figure S11J, Supporting Information). Taken together, these results suggest that USP20 overexpression improves cardiac hypertrophy and dysfunction.

### 2.8 USP20 Ameliorates Cardiac Hypertrophy and Dysfunction by Inhibiting STAT3

**Figure 8:**
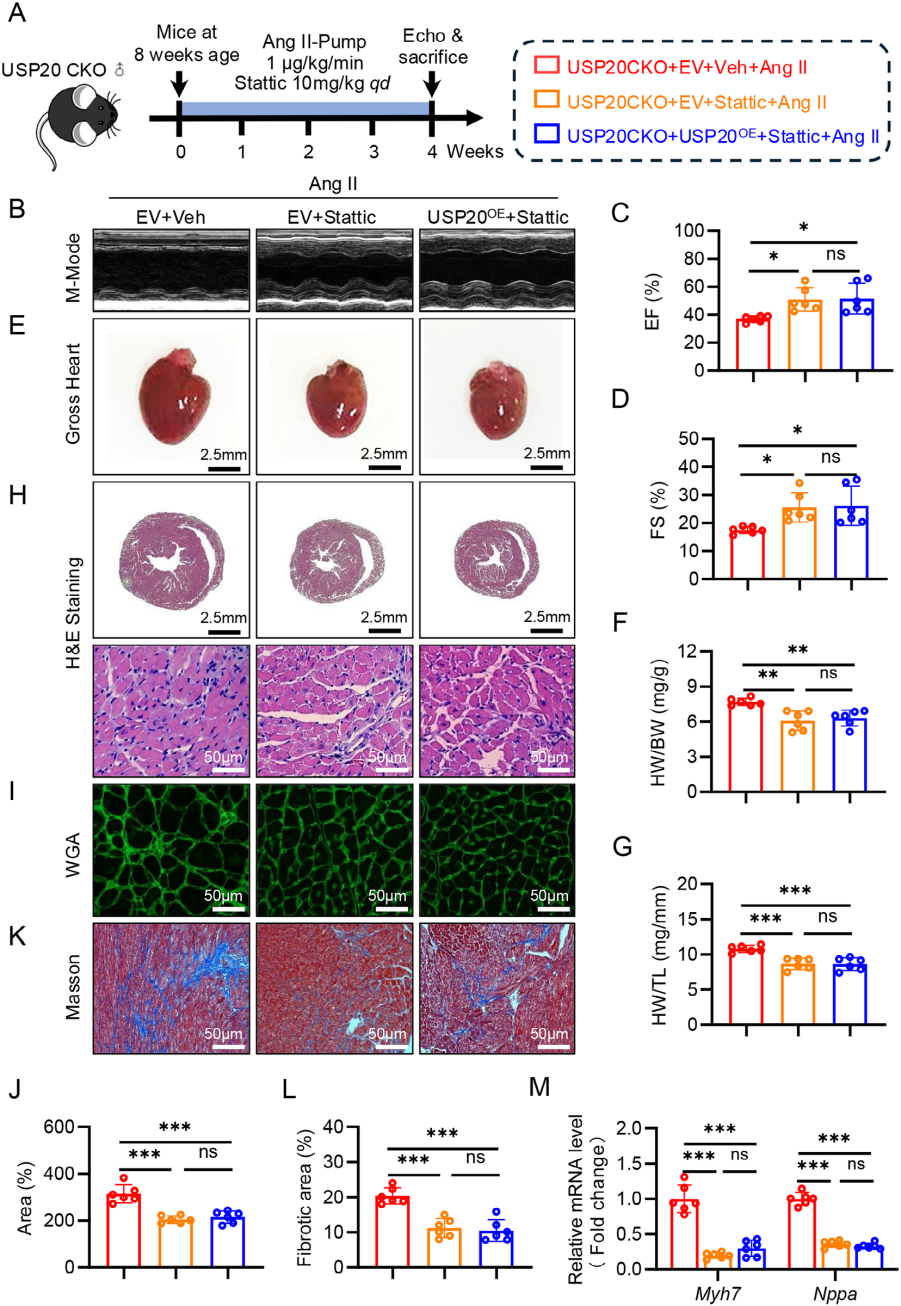
USP20 ameliorates cardiac hypertrophy and dysfunction by inhibiting STAT3. A) USP20 CKO mice were implanted with Ang II‐infused osmotic mini‐pump for 4 weeks. During this period, STAT3 inhibitor stattic was administered every day (10mg kg^−1^, qd). B) Representative M‐mode echocardiographic images from each group in mice. C,D) Echocardiographic analysis of ejection fraction (EF) and fractional shortening (FS). E) Representative images of gross‐heart from each group. F) The ratio of heart weight (HW) to body weight (BW). G) The ratio of heart weight (HW) to tibial length (TL). H) HE stained images of heart sections. Scale bar, 2.5 mm and 50 µm. I,J) Wheat germ agglutinin (WGA) stained images of heart sections. Scale bar, 50 µm (I) and quantitative analysis (J). K,L) Representative masson stained images of myocardial interstitium in heart sections. Scale bar, 50 µm (K) and quantitative analysis (L). M) RT‐qPCR analysis of Myh7 and Nppa in heart tissues. *n* = 6. **p < 0.01, ***p < 0.001, n.s., no significance.

To assess whether USP20 can improve cardiac hypertrophy by regulating STAT3, we administered wild type and USP20 CKO mice with or without STAT3 inhibitor stattic (10mg kg^−1^, i.g.). USP20 CKO mice was injected with AAV9‐USP20 and empty vehicle for 4 weeks to overexpress the level of USP20 in the hearts. Then these mice were implanted with Ang II osmotic pumps to induce cardiac hypertrophy for 4 weeks, and administered with static every day (10mg kg^−1^, i.g., **Figure** **8**A). Initially, we assessed the in vivo inhibitory effects of Stattic on STAT3 activity. Our results demonstrated that Stattic significantly reduced the levels of p‐STAT3 in AngII‐induced hypertrophic hearts in mice, thereby confirming the effective suppression of STAT3 signaling in vivo (Figure S12A,B, Supporting Information). Ang II levels rose in all mice administered with Ang II (Figure S12C, Supporting Information), leading to increased systolic blood pressure (Figure S12D,E, Supporting Information). Stattic did not alter the body weight (Figure S12F, Supporting Information). Cardiac function assessment demonstrated that STAT3 inhibitor stattic protected heart from dysfunction caused by USP20 deficiency, and more importantly, the USP20 expression in the heart failed to ameliorate Ang II‐induced cardiac dysfunction when the stattic was administered (Figure 8B–D; and Table S7, Supporting Information). Similar results were observed for hypertrophic morphology (Figure 8E–G), the cardiomyocyte size (Figure 8H–J), the fibrosis level (Figure 8K,L; Figure S12G,H, Supporting Information), and hypertrophic markers (Figure 8M). These results reveal that USP20 exerts a therapeutic effect on cardiac hypertrophy and dysfunction by inhibiting STAT3. It was also confirmed in vitro that USP20 affects cardiomyocyte hypertrophy by regulating STAT3 (Figure S12I–M, Supporting Information). Therefore, these data demonstrate that USP20 ameliorates cardiac hypertrophy by inhibiting STAT3.

## 3 Discussion

Pathological cardiac hypertrophy can progress to heart failure, a significant global health challenge.^[^ ^45^, ^46^ ^]^ Although β‐adrenergic receptor blockers and renin‐angiotensin‐aldosterone system inhibitors offer some benefit, the incidence of heart failure following hypertrophy remains extensive.^[^ ^47^ ^]^ Understanding the molecular mechanisms underlying cardiac hypertrophy is therefore essential for developing better therapeutic interventions. Deubiquitination, an important post‐translational modification mediated by deubiquitinating enzymes (DUBs), modulates signal transduction and prevents substrates degradation by altering ubiquitin linkages.^[^ ^48^, ^49^ ^]^ Elucidating the regulatory roles and mechanisms of DUBs in cardiac hypertrophy can provide novel therapeutic strategies. Recent studies have highlighted the involvement of DUBs such as USP25,^[^ ^19^ ^]^ JOSD2,^[^ ^20^ ^]^ and USP28^[^ ^21^, ^50^ ^]^ in cardiovascular diseases. USP25 and JOSD2 have been shown to exert cardioprotective effects by stabilizing SERCA2a, a key regulator of calcium homeostasis, thereby ameliorating pathological hypertrophy.^[^ ^19^, ^20^ ^]^ This suggests that USP25 operates through substrate‐specific mechanism, emphasizing the diverse roles of DUBs in maintaining cardiomyocyte function. Our findings highlight a protective role of USP20 by targeting STAT3 in hypertrophic models induced by TAC and Ang II, in contrast to USP28, which has been reported to exacerbate pathological cardiac hypertrophy in similar settings through mechanisms such as TRIM21‐mediated ROS accumulation.^[^ ^50^ ^]^ This apparent contradiction underscores the complexity of DUB function in cardiovascular diseases and suggests that substrate specificity may play a pivotal role in determining their effects on downstream signaling pathways. A deeper understanding of the molecular networks regulated by these enzymes will be critical for developing targeted therapies. Future studies should aim to identify shared and unique substrates of these DUBs and elucidate the spatiotemporal regulation of their activity in different cardiac pathologies.

In this study, we identified USP20 specifically enriched in cardiomyocytes, as a crucial factor down‐regulated in pathological cardiac hypertrophy. Although USP20 has been extensively studied in pathological processes, particularly in cancer biology.^[^ ^22^, ^23^, ^51^ ^]^ Its function in cardiac protection has only been explored recently.^[^ ^25^, ^26^, ^52^ ^]^ Consistent with our findings, recent study indicated that constitutive knockout of USP20 exacerbates pressure overload‐induced cardiac hypertrophy in TAC model.^[^ ^52^ ^]^ We employ cardiomyocyte‐specific USP20 knockout and overexpressed mice to elucidate the function and mechanisms of USP20 in vivo. Our data substantiate the protective role of USP20 in pathological cardiac hypertrophy, and present novel translational potentials by targeting USP20 and its downstream axis with small molecule inhibitor.

The function of DUBs is related to their substrate.^[^ ^53^ ^]^ In this study, we identified STAT3 as a direct substrate of USP20 in myocardial hypertrophic myocardium and mouse‐derived HL‐1 cells. STAT3 is crucial for cellular survival, growth, sarcomere architecture, energy dynamics, and metabolism.^[^ ^54^, ^55^ ^]^ Since activated STAT3 promotes heart failure,^[^ ^56^, ^57^ ^]^ STAT3 has become a pivotal therapeutic target for heart failure, with gene therapy approaches showing promising results in patients with cardiac hypertrophy.^[^ ^58^ ^]^ However, STAT3 regulates numerous biological processes across almost all human cells and organs, making it an unsuitable direct therapeutic target.^[^ ^59^ ^]^ Constant activation of STAT3 induces hypertrophy and inflammation in the heart and other organs, such as the gut, and promote cellular de‐differentiation, potentially leading to cancer.^[^ ^60^ ^]^ But meanwhile, directly reducing or blocking STAT3 below a certain threshold can lead to impaired cardiac vasculature, increased fibrosis, and heart failure.^[^ ^55^ ^]^ A new therapy targeting cell‐type‐specific STAT3 regulation becomes a challenging goal for direct pharmacological approaches. In this research, we identified an unannotated cardiomyocyte‐specific USP20‐STAT3 axis, highlighting its role in regulation of cardiac hypertrophy (***Structured Graphical Abstract***).

The effect of ubiquitination is mainly determined by the type of ubiquitin chain attached to the substrate.^[^ ^53^ ^]^ Different patterns of deubiquitination on substrate proteins impart distinct functional roles to DUBs. Generally, USP20 modulates substrate stability by regulating K48‐linked ubiquitination, while its role in K63‐linked ubiquitination or other ubiquitin chains are rarely reported.^[^ ^61^, ^62^ ^]^ K63‐linked ubiquitination serves as a molecular scaffold for protein‐protein interactions, playing a crucial role in activating protein kinase signaling, receptor endocytosis, protein trafficking, and DNA damage repair.^[^ ^63^, ^64^ ^]^ It has been shown that K63‐linked polyubiquitination of STAT3 influences its phosphorylation and nuclear translocation, thereby modulating transcriptional activity of STAT3.^[^ ^35^, ^36^ ^]^ Our data demonstrate that USP20‐mediated deubiquitination of STAT3 specifically targets K63‐linked ubiquitin chains. Using ubiquitylome analysis and validation, we identified lysine 177 (K177) in the coiled‐coil domain (CCD) of STAT3 as the key site for USP20 binding. This domain regulates STAT3 phosphorylation and nuclear translocation through allosteric modulation of the SH2 domain.^[^ ^43^ ^]^ It is noteworthy that K177 is in the CCD of STAT3, and therefore we speculated that ubiquitination at K177 affects STAT3's interaction with its target gene promoters, providing novel insights into its regulatory mechanisms.

Accumulating evidence suggests that STAT3 also acts as a transcriptional suppressor in certain biological process.^[^ ^28^, ^65^ ^]^ One of the mechanisms by which STAT3 inhibits gene expression is the acetylation of the K685 site in the SH2 domain, which is crucial for the binding between STAT3 and DNA methyltransferase 1, leading to the methylation and silencing of different promoters.^[^ ^65^ ^]^ However, the transcriptional regulation of STAT3 in cardiomyocytes remains poorly understood. Our results provide the first evidence that STAT3 directly binds to the promoter region of *Carm1*, and inhibits its transcription (***Structured Graphical Abstract***). Notably, this inhibitory effect was significantly enhanced by inhibition of USP20 or diminished by overexpression of USP20. Interestingly, this study demonstrates a previously unannotated functional link between STAT3 and CARM1 in the pathogenesis of pathological myocardial hypertrophy. Whether other transcription factors involve in the USP20‐STAT3‐CARM1 axis will be explored in future studies.

There are some limitations to our study. First, our studies have confirmed that STAT3 is a substrate of USP20; however, we cannot exclude the possibility that USP20 may regulate other substrate proteins to mediate cardiomyocyte hypertrophy. Further investigations are needed to elucidate the other mechanisms by which cardiomyocyte‐derived USP20 regulates cardiac hypertrophy. Second, while protein levels are modulated by gene expression and post‐translational modifications (PTMs),^[^ ^17^ ^]^ the transcription factors that regulates USP20 expression remain largely unknown. Our data show that USP20 expression diminishes no later than 3 hours following Ang II treatment, suggesting the involvement of PTMs. Ubiquitination, a key PTM regulating protein degradation, may also contribute to alter USP20 protein level. Additional research is necessary to investigate the involvement of PTMs in the USP20‐mediated pathogenesis of cardiac hypertrophy.

## 4 Conclusion

In summary, we elucidate that cardiomyocyte‐enriched USP20 regulates K63‐linked ubiquitination of STAT3. We demonstrate that the cardiomyocyte‐specific USP20‐STAT3‐CARM1 axis plays a protective role in cardiac hypertrophy, suggesting that targeting USP20 with cardiac‐specific gene therapy could be a promising strategy for treating cardiac hypertrophy.

## 5 Experimental Section

A detailed description of the materials and methods is provided in the Supporting Information.

### Study Approval

All experiments involving live animals adhered strictly to the Guide for the Care and Use of Laboratory Animals (U.S. National Institutes of Health, NIH publication no. 85‐23, revised 1996). All experimental procedures were conducted under the approval of the Institutional Animal Care and Use Committee (IACUC) of the Second Affiliated Hospital, Zhejiang University School of Medicine. Human heart samples: Left ventricular (LV) tissues were taken from patients with terminal‐stage heart failure indicated for heart transplantation. In brief, the patient's heart was removed at the time of transplantation, and LV tissue was subsequently dissected and snap‐frozen. LV samples were used from healthy hearts that were not implanted to serve as controls. All experimental protocols involving patients were approved by the Ethics Committee of the Second Affiliated Hospital Zhejiang University School of Medicine.

### Statistical Analysis

The normal distribution of each data was evaluated using the Shapiro‐Wilk test. The data between the two groups with normal distribution were compared using a two‐sided unpaired Student's t‐test. If not, the Mann‐Whitney U test was employed as an alternative. For comparison among multiple groups with normal distribution, either one‐way ANOVA or two‐way ANOVA analysis of variance was utilized followed by Bonferroni's post hoc test. Otherwise, the Kruskal‐Wallis nonparametric test was applied. All experimental data were statistically analyzed and visualized using GraphPad Prism 8.0 software (San Diego, CA, USA) with the results presented as mean ± standard deviation. Statistical significance was defined as a P value less than 0.05 and denoted in the figures as* p < 0.05, ** p < 0.01, and *** p < 0.001.

## Conflict of Interest

The authors declare no conflict of interest.

## Author Contributions

L.Z. and S.D. contributed equally to this work. L.F.Z. and S.S.D. designed and carried out the most experiments, performed statistical analysis, and generated the figures. L.F.Z. and S.S.D. performed most animal assay supervised by J.H.C. and D.L.Y.; S.S.D. assisted L.F.Z. for primary cardiomyocyte preparation and part of cell assays; F.Y., Q.Y.G., Y.C.Z., J.‐S.D., and Z.Y.L. helped with the experiments; Z.X.T. and F.Z.G. assisted S.S.D. for the single‐cell sorting and helped with image data analysis; D.R.C. assisted L.F.Z. for Co‐IP and CUT&Tag experiments. L.Y.H. provided technical assistance to L.F.Z. and S.S.D.; L.F.Z., S.S.D., and D.L.Y. conceived the study; L.F.Z., S.S.D., and Q.Y.G. wrote the initial manuscript; D.L.Y. and J.H.C. provided important discussions and essential revisions. G.P.S., X.Y.H., J.H.C., J.A.W., and D.L.Y. supervised the study; J.A.W. and D.L.Y. designed and oversaw the study. All authors have read and approved the article.

## Supporting information



Supporting Information

Supplemental Table 1

## Data Availability

The data that support the findings of this study are available from the corresponding author upon reasonable request.
