# Supplementary material for: Cardiomyocyte‐Enriched USP20 Ameliorates Pathological Cardiac Hypertrophy by Targeting STAT3 Deubiquitination
Source: Adv Sci (Weinh). 2025 Apr 7;12(23):2416478. doi: 10.1002/advs.202416478 (PMC12199517; doi:10.1002/advs.202416478)
Supplement: Supplementary file 1 — Supporting Information [file ADVS-12-2416478-s001.docx]

**Cardiomyocyte-Enriched USP20 Ameliorates Pathological Cardiac Hypertrophy by Targeting STAT3 Deubiquitination**

*Lingfeng Zhong, ^1,2,3†^ Shanshan Dai,^4†^ Fan Yu, ^1,2,3^Guo-Ping Shi,^5^ Qinyan Gong,^1,2,3^ Yucong Zhang,^6^ Jingsi Duan, ^1,2,3^ Zhengyin Lou,^1,2,3^ Zhixuan Tang,^6^ Fuzhe Gong,^1,2,3^ Derong Chen,^1,2,3^ Liya Hou,^1,2,3^ Xinyang Hu, ^1,2,3^Jinghai Chen,^1,2,3,7^* Jian’an Wang,^1,2,3^* and Deling Yin^1,2,3^**

^1^ Department of Cardiology of the Second Affiliated Hospital, Zhejiang University School of Medicine, Hangzhou 310009, China
^2^ State Key Laboratory of Transvascular Implantation Devices, Hangzhou 310009, China
^3^ Heart Regeneration and Repair Key Laboratory of Zhejiang Province, Hangzhou 310009, China

^4^ The Key Laboratory of Emergency and Disaster Medicine of Wenzhou, Department of Emergency, The First Affiliated Hospital of Wenzhou Medical University, Wenzhou 325000, China.

^5^ Department of Medicine, Brigham and Women's Hospital, Harvard Medical School, 77 Avenue Louis Pasteur, NRB-7, Boston, MA 02115, USA.

^6^ Department of Cardiology of First Affiliated Hospital of Wenzhou Medical University, Wenzhou 325000, China

^7^ Institute of Translational Medicine, Zhejiang University School of Medicine, Hangzhou 310029, China

^†^ *Lingfeng Zhong* and *Shanshan Dai* contributed equally to the study.

*Corresponding authors.

**Correspondence to**

Deling Yin, Jian’an Wang, Jinghai Chen. Email address: [yindl@zju.edu.cn](mailto:yindl@zju.edu.cn) (Deling Yin), wangjianan111@ zju.edu.cn (Jian’an Wang), [Jinghaichen@zju.edu.cn](mailto:Jinghaichen@zju.edu.cn) (Jinghai Chen).

**Supplementary Figures and legends**

**
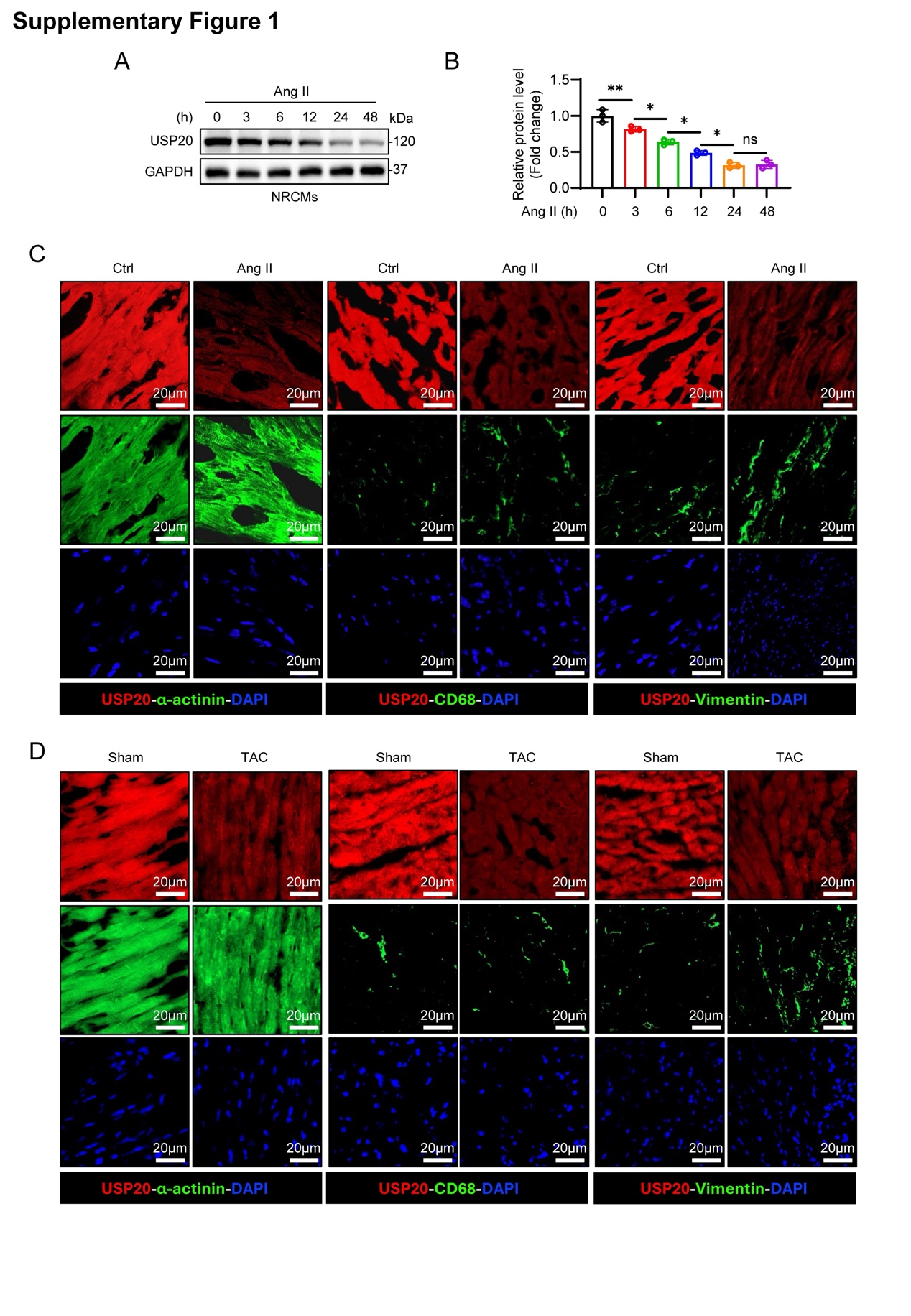
**

**Figure S1.** A-B) Neonatal rat cardiomyocytes (NRCMs) were incubated with Ang II at 1 μM. A) Representative immunoblot of USP20 expression and quantitative analysis (B)*.* (*n* = 3). *p < 0.05, **p < 0.01, n.s., no significance. C-D) Representative immunofluorescence staining for USP20 (red), α-actinin for cardiomyocyte (green), CD68 for macrophage (green), vimentin for fibroblast (green) in the heart sections from Ang II administration-induced (C) or TAC-induced cardiac hypertrophy of micec (D). Merged images (yellow) showing co-localization. Results are representative of three independent experiments.


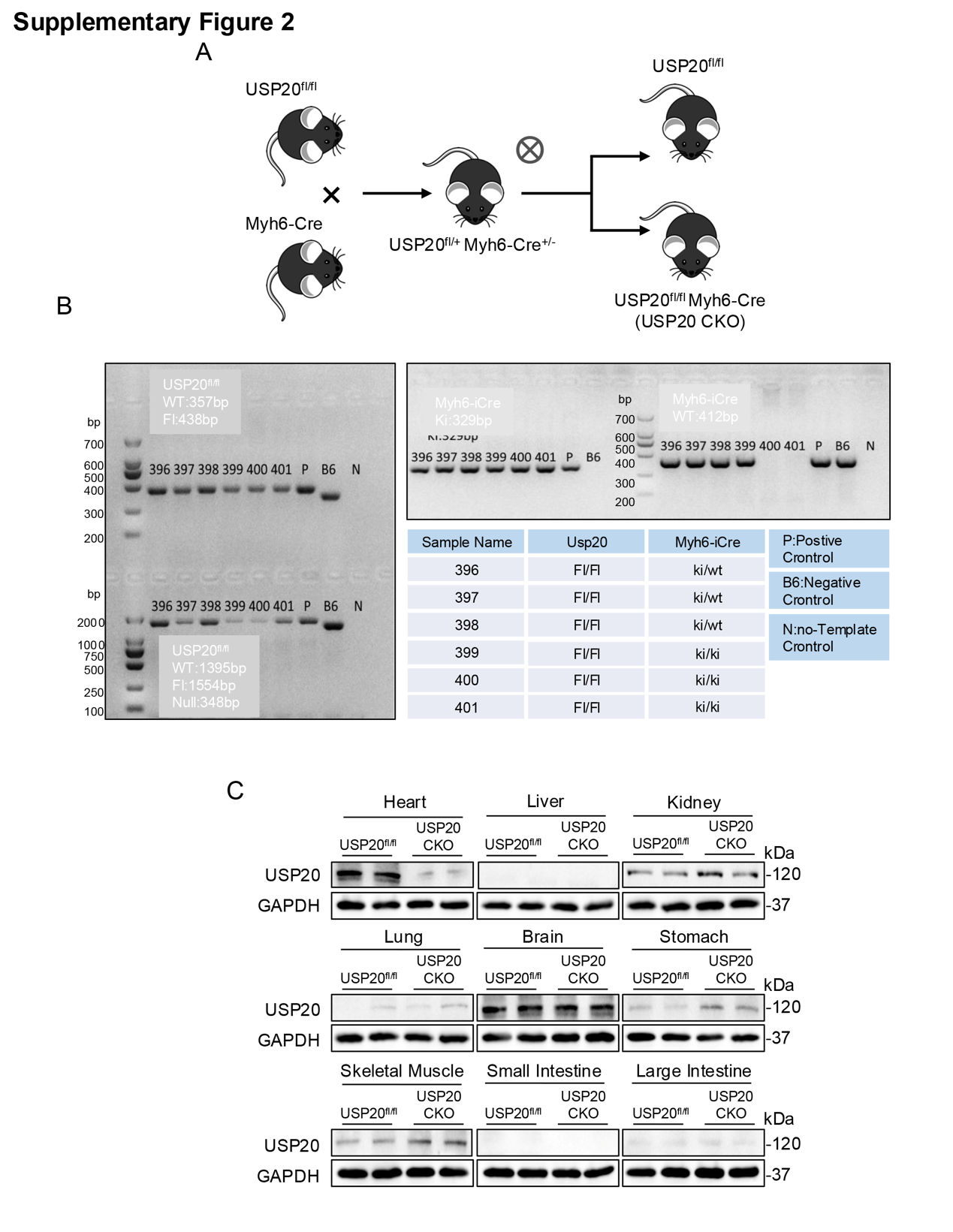


**Figure S2.** A) Schematic diagram of the strategy for the generation of cardiomyocyte-specific USP20 knockout mice (USP20 CKO). B) The PCR primers of Usp20 (WT: 357 bp; Fl: 438 bp) and Myh6-cre (WT: 412 bp; KI 329 bp) were used respectively to identify the genotype of mice. (P: positive control; B6: Negative control; N: No-template control). C) Representative immunoblotting of USP20 in various organs and tissues from USP20^fl/fl^ and USP20 CKO mice.


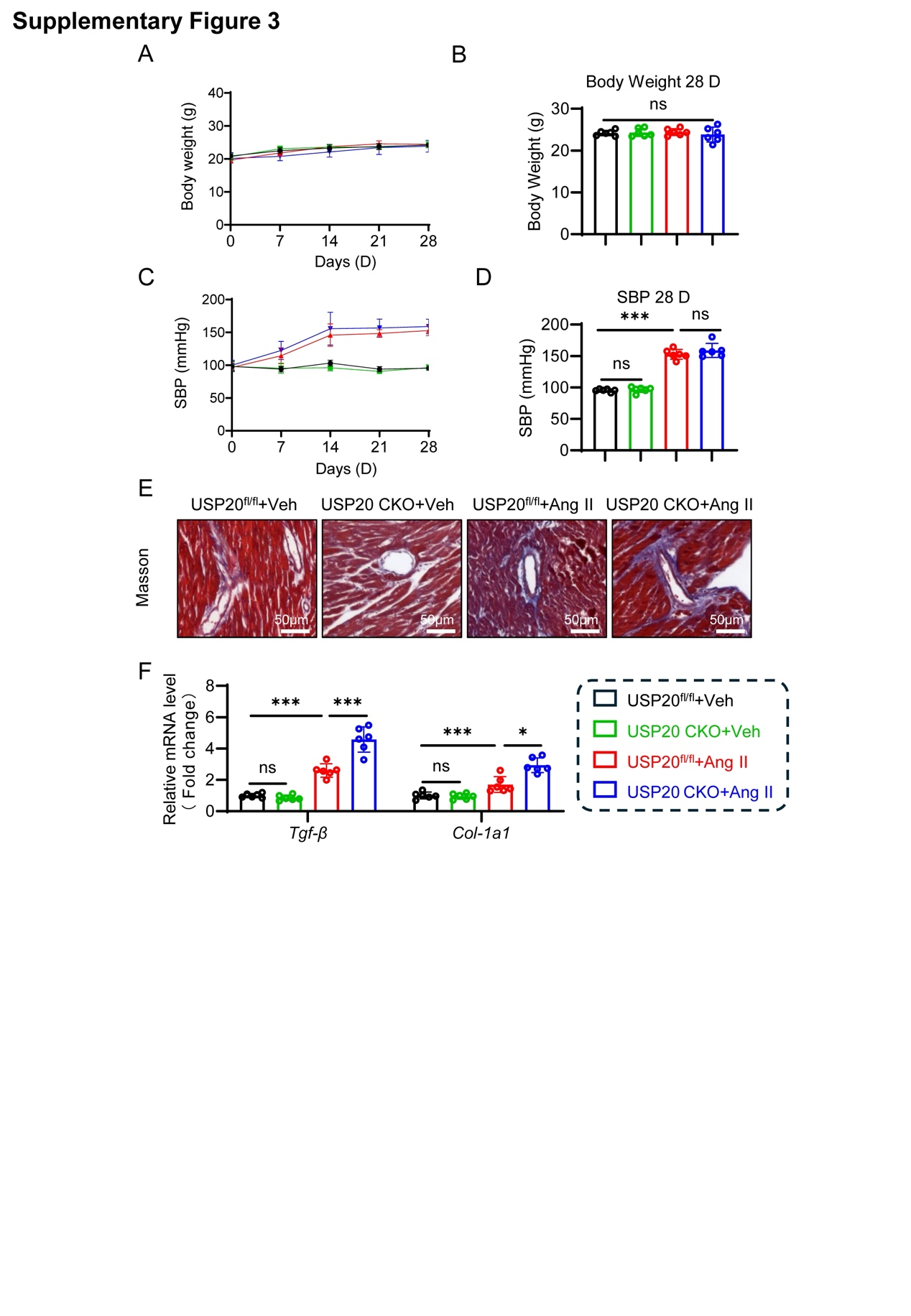


**Figure S3.** A-B) Body weight (BW) of mice during studies of time periods (A) and at day 28 (B). (*n* = 6). ns., no significance. C-D) Mice systolic blood pressure (SBP) during studies of time periods (C) and at day 28 (D)*.* (*n* = 6). ns., no significance. E) Representative masson stained images of perivascular regions in the heart sections. Scale bar, 50 μm. F) RT-qPCR analysis of *Tgf-β* and *Col-1a1* in the heart tissues. (*n* = 6). *p < 0.05, ***p < 0.001, ns., no significance.


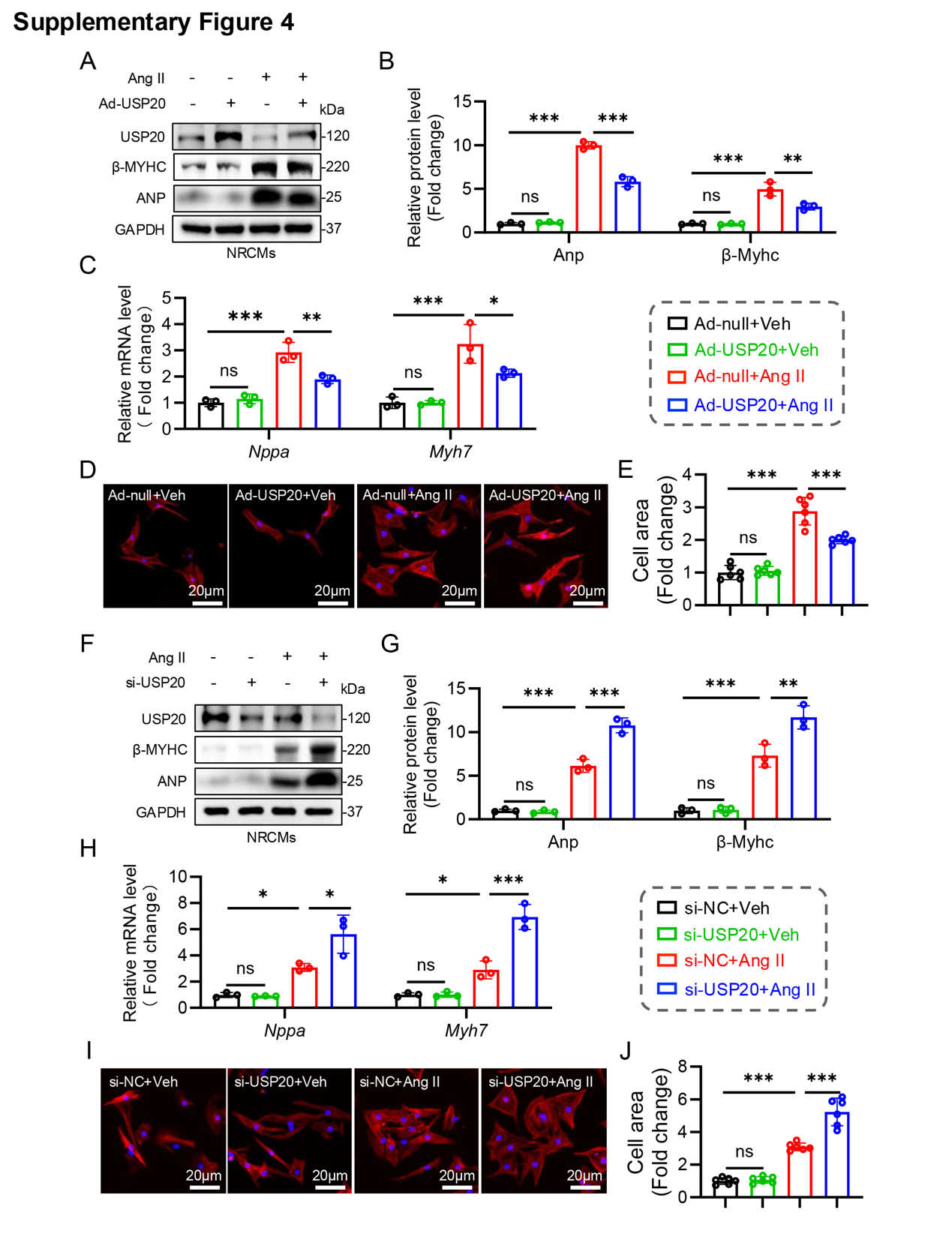


**Figure S4.** A-G) NRCMs were infected with either adenovirus (Ad) encoding USP20 (Ad-USP20) at MOI of 50 or Ad-null as control, followed by incubation with Ang II at 1 μM for 24 h. A-B) Representative immunoblot of USP20, β-myosin heavy chain (β-MYH. C), and atrial natriuretic peptide (ANP) (A) and quantification analysis (B). (*n* = 3). **p < 0.01, ***p < 0.001, ns., no significance. C) RT-qPCR analysis of *Nppa* and *Myh7* in NRCMs. (*n* = 3). *p < 0.05, **p < 0.01, ***p < 0.001. D-E) The surface area of NRCMs was examined by TRITC-labeled rhodamine phalloidin staining (D) and quantitative analysis (E)*.* (*n* = 6). ***p <0.001, ns., no significance. F-J) NRCMs were transfected with siRNAs of si-NC (negative control) or si-USP20 following with Ang II stimulation at 1 μM for 24 h. Representative immunoblotting of USP20, β-MYHC and ANP (F) and densitometric quantification (G)*.* (*n* = 3). **p < 0.01, ***p < 0.001. H) RT-qPCR analysis of *Nppa* and *Myh7* in NRCMs. (*n* = 3). *p < 0.05, ***p < 0.001. I-J) The TRITC-labeled rhodamine phalloidin staining (I) and quantitative analysis (J)*.* (*n* = 6). ***p < 0.001, ns., no significance.


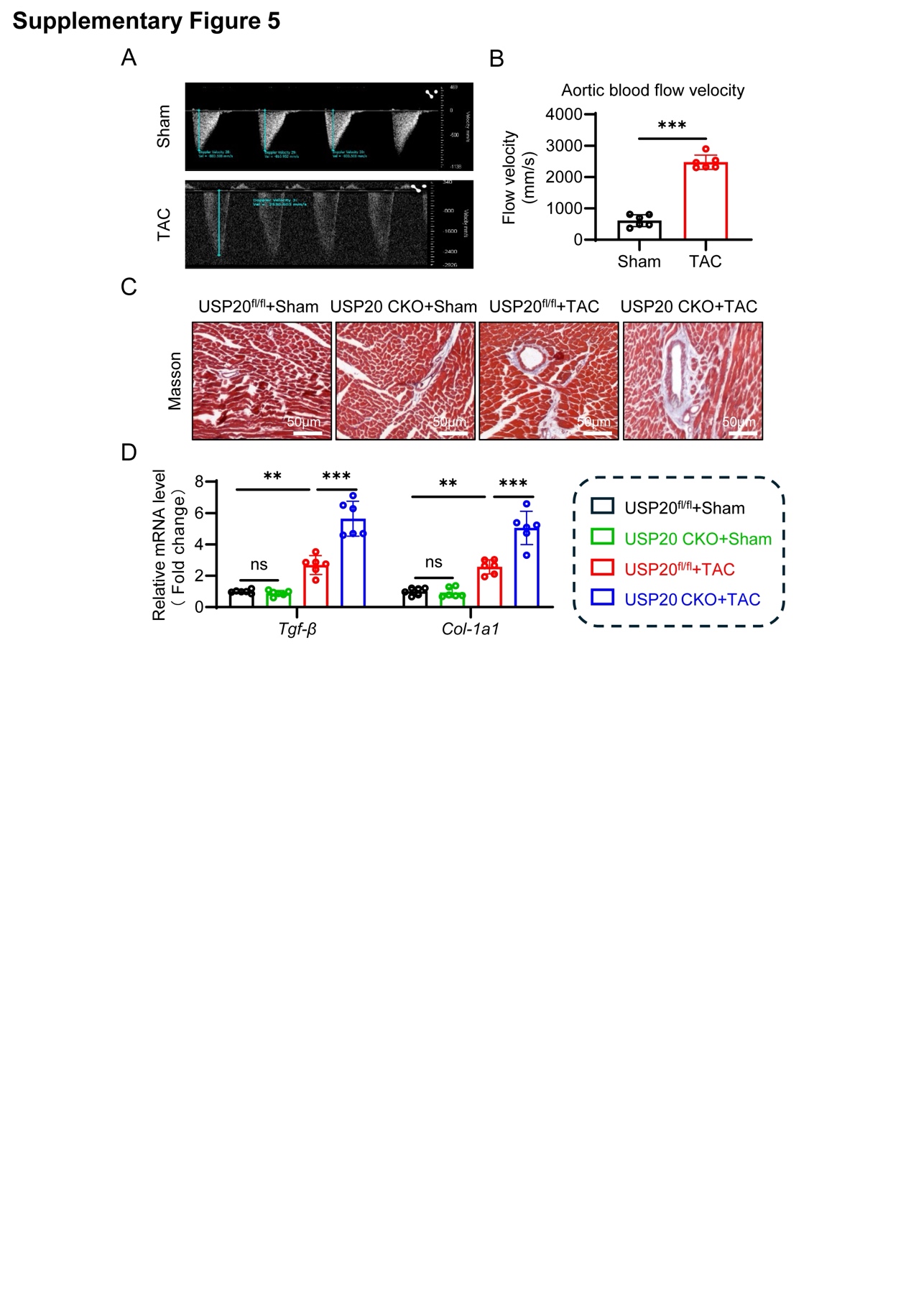


**Figure S5.** A-B) Representative flow doppler ultrasound images of the aortic arch (A) and the quantitative analysis (B) of transverse aortic flow velocity from Sham or TAC-induced mice. (*n* = 6). ***p < 0.001. C) Representative masson stained images of perivascular regions in the heart tissue sections from TAC-induced cardiac hypertrophy. Scale bar, 50 μm. D) RT-qPCR analysis of *Tgf-β* and *Col-1a1* in heart tissues as from (C)*.* (*n* = 6). **p < 0.01, ***p < 0.001, ns., no significance.


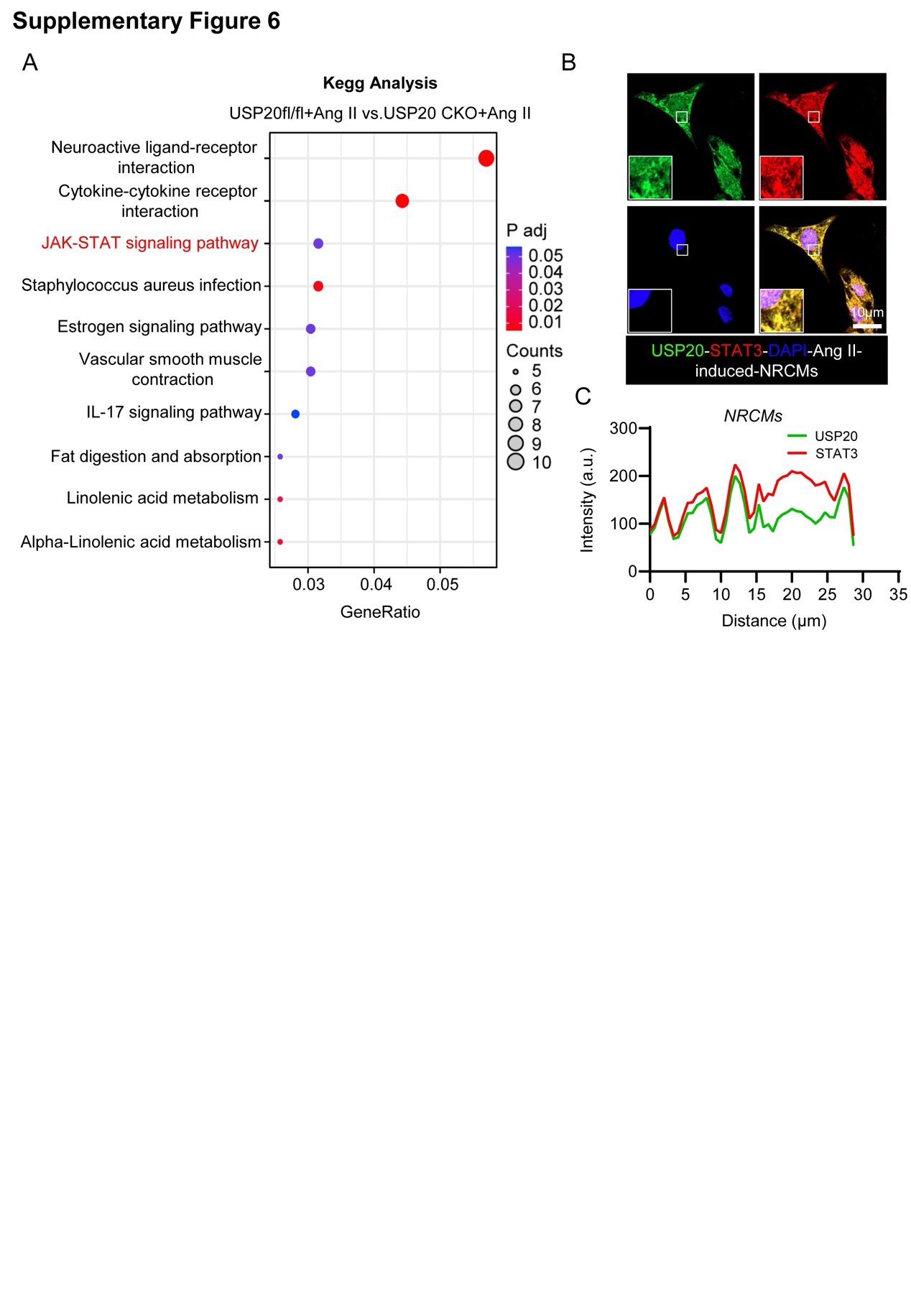


**Figure S6.** A) KEGG enrichment analysis of transcriptome of the heart tissues from Ang II- induced USP20 CKO mice and USP20^fl/fl^ mice. B-C) Co-localization of endogenous USP20 (green) and STAT3 (red) in Ang II-treated NRCMs (B) and qualitative analysis (C)*.* Results are representative of three independent experiments.


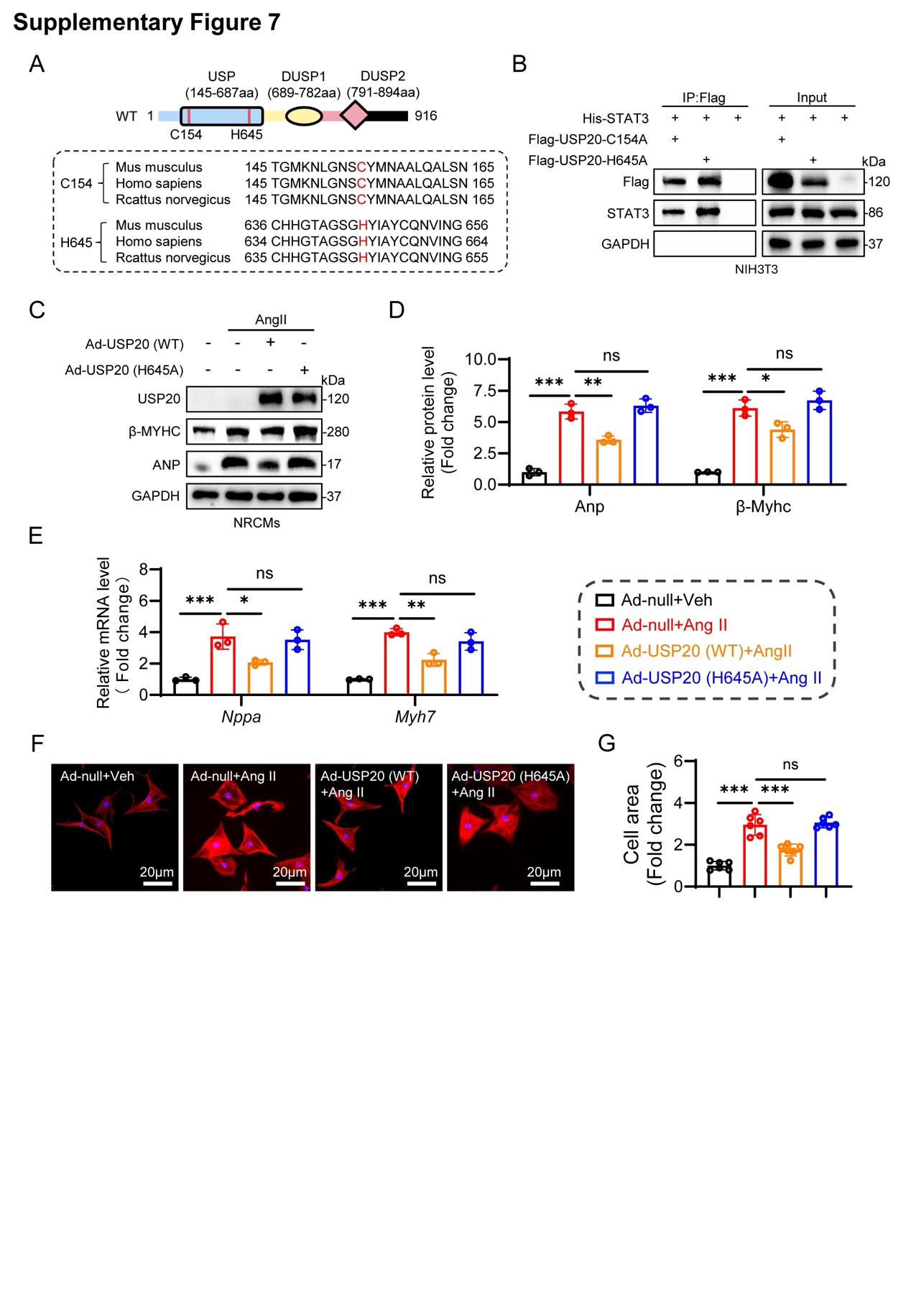


**Figure S7.** A) Homology analysis of the active sites C154 and H645 of USP20. B) NIH3T3 cells were co-transfected His-STAT3 with either Flag-USP20^C154A^ or Flag-USP20^H645A^ plasmids and then co-immunoprecipitated. Exogenous normal or mutated USP20 was immunoprecipitated by anti-Flag antibody. C-G) NRCMs were infected with Ad-USP20 (WT) or Ad-USP20 (H645) at MOI of 50 and Ad-null as control, followed by Ang II stimulation at 1 μM for 24 h. C-D) Representative western blotting (C) and densitometric quantification (D) of USP20, β-Myhc and Anp in NRCMs. (*n* = 3). ns., no significance. *p < 0.05, **p < 0.01, ***p < 0.001. E) RT-qPCR analysis of *Nppa* and *Myh7* in NRCMs. (*n* = 3). ns., no significance. **p *<*0.01, ***p *<* 0.001. F-G) The TRITC-labeled rhodamine phalloidin staining (F) and quantitative analysis (G). (*n* = 6). *p < 0.05, **p < 0.01, ***p < 0.001, ns., no significance.


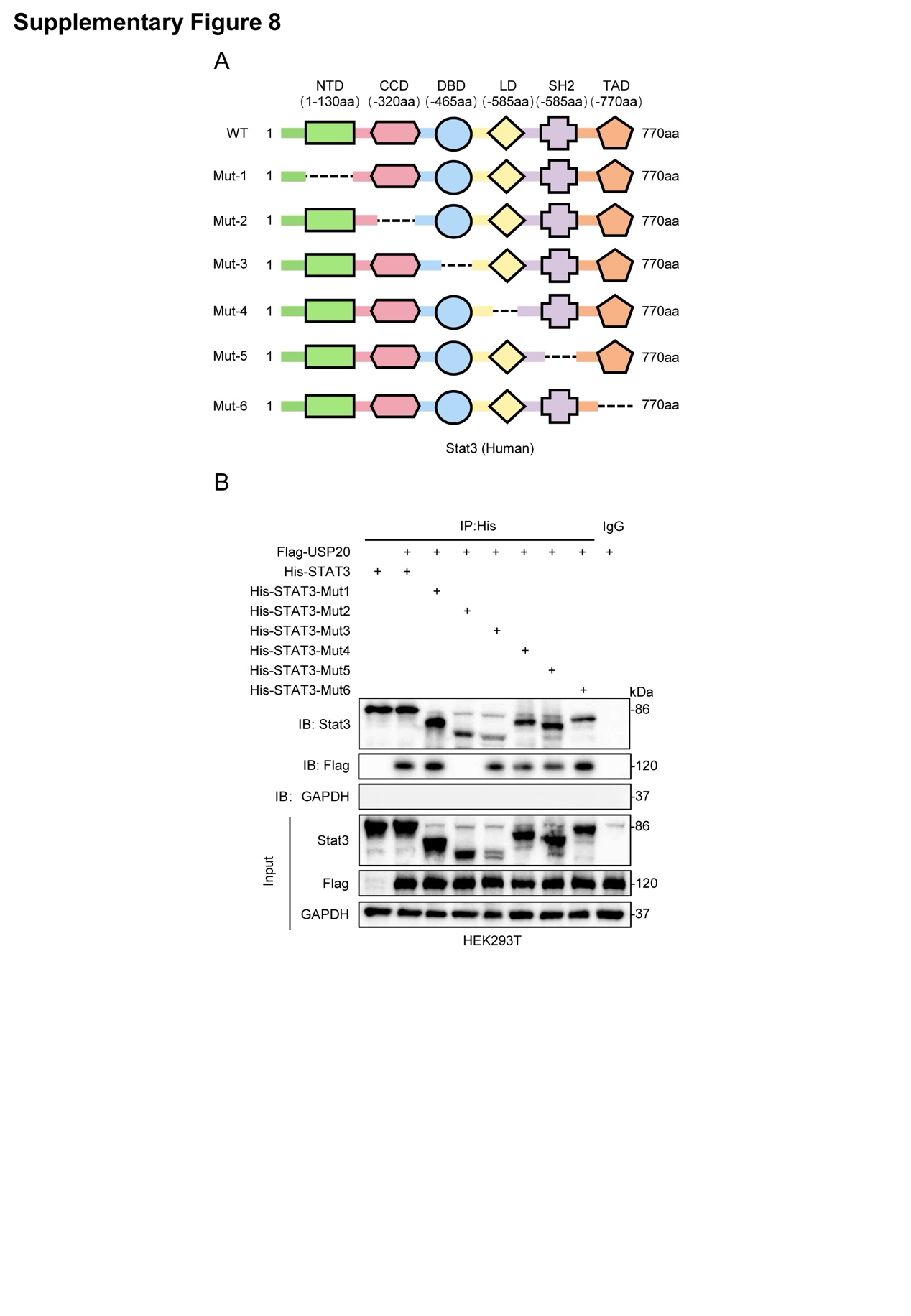


**Figure S8.** A) Schematic illustration of the STAT3 domain deletion construct used in (B). B) HEK293T cells were co-transfected Flag-USP20 with either overexpression plasmids of His-WT-STAT3 or different mutations of His-Mut-STAT3 and then co-immunoprecipitated. Exogenous normal or mutated STAT3 was immunoprecipitated by anti-His antibody.


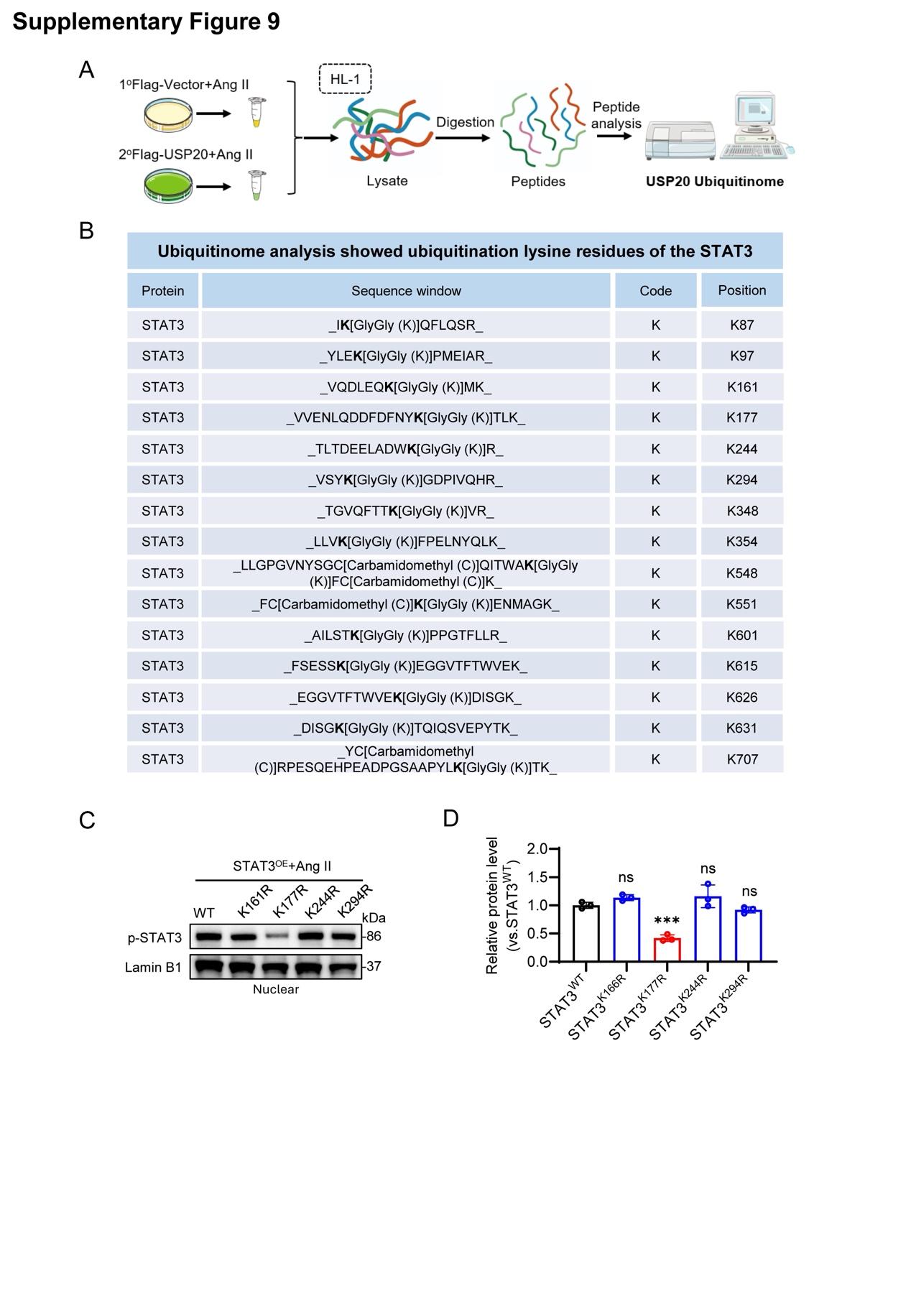


**Figure S9.** A) Schematic illustration of the ubiquitylome analysis. HL-1 cells were transfected with Flag-vector or Flag-USP20 and then incubated with Ang II at 1 μM for 24 h. The proteins were digested to peptide, and then subjected to LC-MS/MS analysis. B) The table showed the USP20- regulated ubiquitination lysine residues of STAT3. C-D) HL-1 cells were transfected with plasmids of WT-STAT3 and different mutations of Mut-STAT3 followed stimulation with Ang II at 1μmol for 24 h. Representative western blotting (C) for p-STAT3 and Lamin B1 in nuclear and the quantitative analysis (D). Lamin B1 was used as loading control. (*n* = 3). ***p < 0.001, ns., no significance.


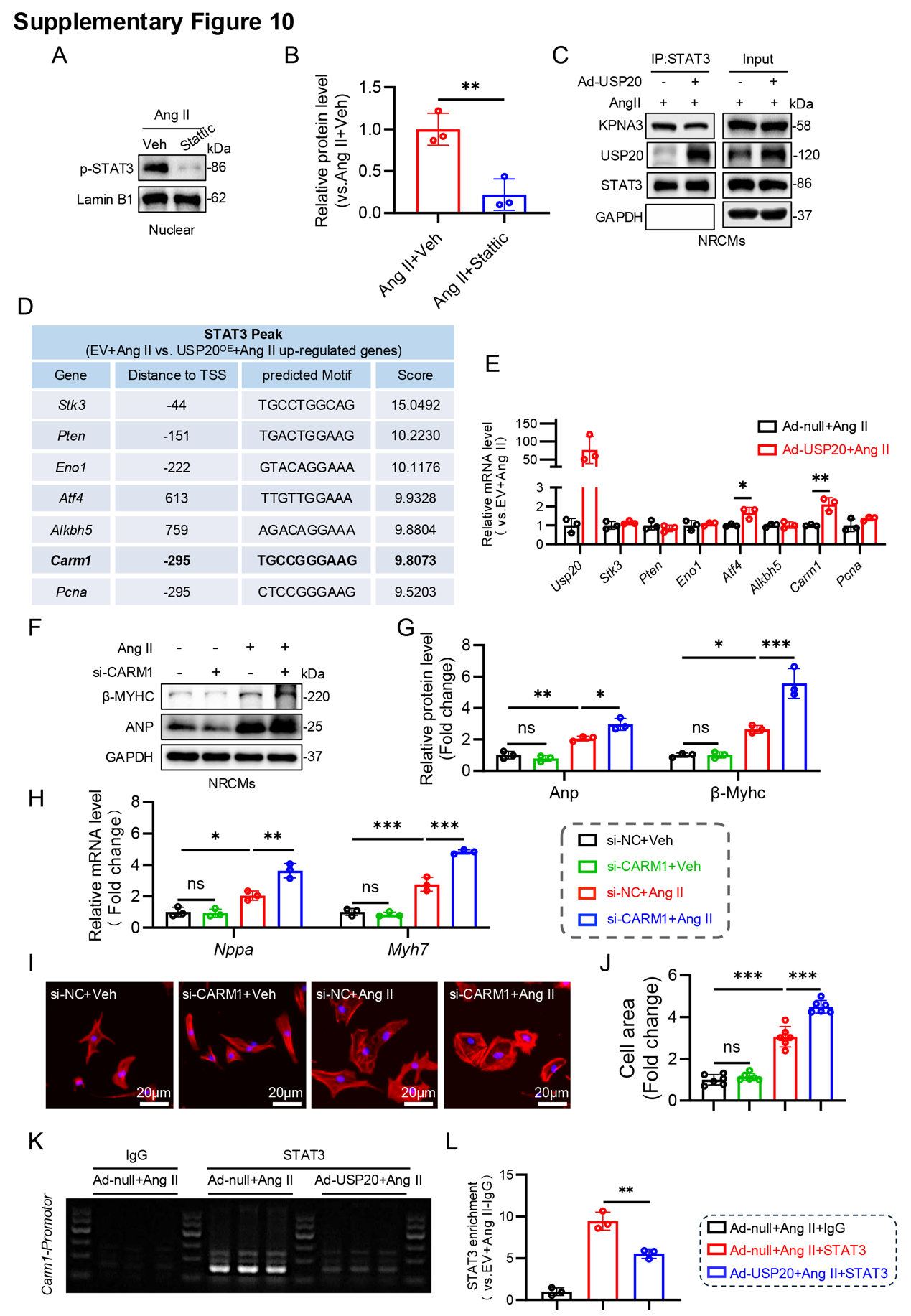


**Figure S10.** A-B) NRCMs were incubated with STAT3 inhibitor stattic at 10 μM for 1 h and then stimulation with Ang II at 1 μM for 24 h. Representative immunoblotting (A) for p-STAT3 and Lamin B1 in nuclear and quantitative analysis (B)*.* Lamin B1 was used as loading control. (*n* = 3). **p < 0.01. C) Immunoprecipitation of KPNA3 and STAT3 in Ang II-treated NRCMs infected with Ad-USP20 at MOI of 50 or Ad-null. D) De novo motif analysis identified from STAT3 peaks. The table showing that the genes related to cardiovascular pathophysiology. E) RT-qPCR analysis of corresponding genes listed in (D) in NRCMs infected with Ad-USP20 or Ad-null. (*n* = 3). *p <0.05, **p <0.01. F-J) NRCMs were transfected with siRNAs of si-NC (negative control) or si-CARM1 following with Ang II stimulation at 1 μM for 24 h. F-G) Representative immunoblotting of β-Myhc and Anp (F) and densitometric quantification (G)*.* (*n* = 3). *p < 0.05, **p < 0.01, ***p < 0.001. H) RT-qPCR analysis of *Nppa* and *Myh7* in NRCMs. (*n* = 3). *p < 0.05, **p < 0.01, ***p < 0.001. I-J) The TRITC-labeled rhodamine phalloidin staining (I) and quantitative analysis (J)*.* (*n* = 6). ***p < 0.001, ns., no significance. K) CUT&Tag was performed on Ad-Null or Ad-USP20-infecteded NRCMs to verify STAT3 binding to the promoter regions of the Carm1 gene. L) CUT&Tag-qPCR assay of the binding of STAT3 at Carm1 promoter regions. (*n* = 3). **p < 0.01.


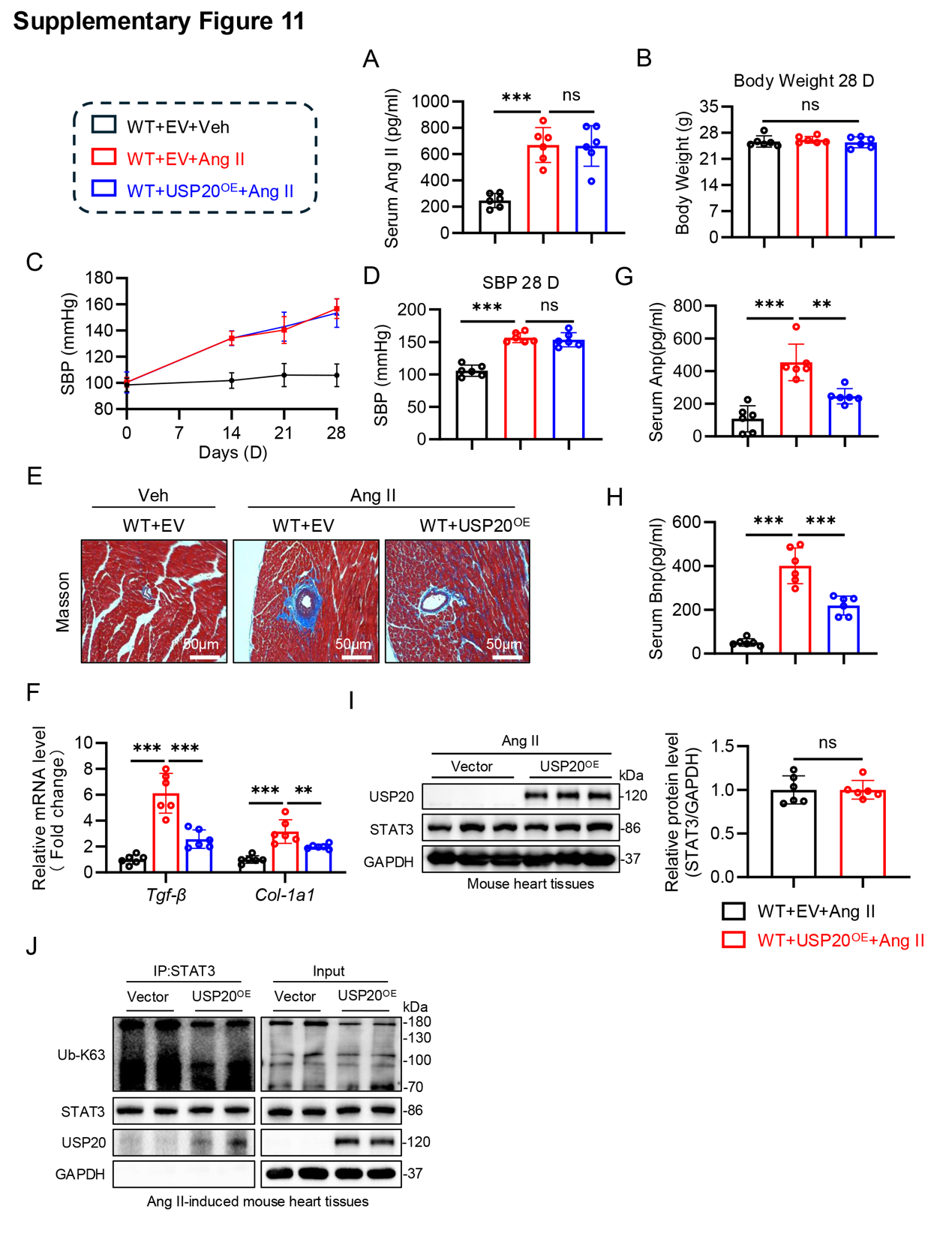


**Figure S11.** A-H) WT mice were injected with AAV9 cardiomyocyte-specific overexpressing USP20 (USP20^OE^) or empty vector (EV) (2 x 10^11^ v.g., i.v.). After 4 weeks, the mice were induced to cardiac hypertrophy by Ang II and then harvested the heart tissues. A) Serum Ang II level was detected by Ang II ELISA kit. B) Body weight (BW) of mice at day 28. (*n* = 6). ***p <0.001, ns., no significance. C-D) Mice systolic blood pressure (SBP) during study (C) and at day 28 (D)*.* (*n* = 6). ns., no significance. E) Representative masson stained images of perivascular regions in heart sections. Scale bar, 50 μm. F) RT-qPCR analysis of *Tgf-β* and *Col-1a1*in heart tissues. (*n* = 6). **p < 0.01, ***p < 0.001. G) Serum Anp level was detected by Anp ELISA kit. (*n* = 6). **p < 0.01, ***p < 0.001. H) Serum Bnp level was detected by Bnp ELISA kit. (*n* = 6). ***p < 0.001. I) Representative immunoblotting of USP20 and STAT3 and quantitative analysis of STAT3. (*n* = 6). ***p < 0.001, ns., no significance. J) STAT3 was immunoprecipitated. Ubiquitinated STAT3 was detected by immunoblotting with an Ub-K63 antibody to clarify the K63 ubiquitination level of STAT3 regulated by USP20.

**
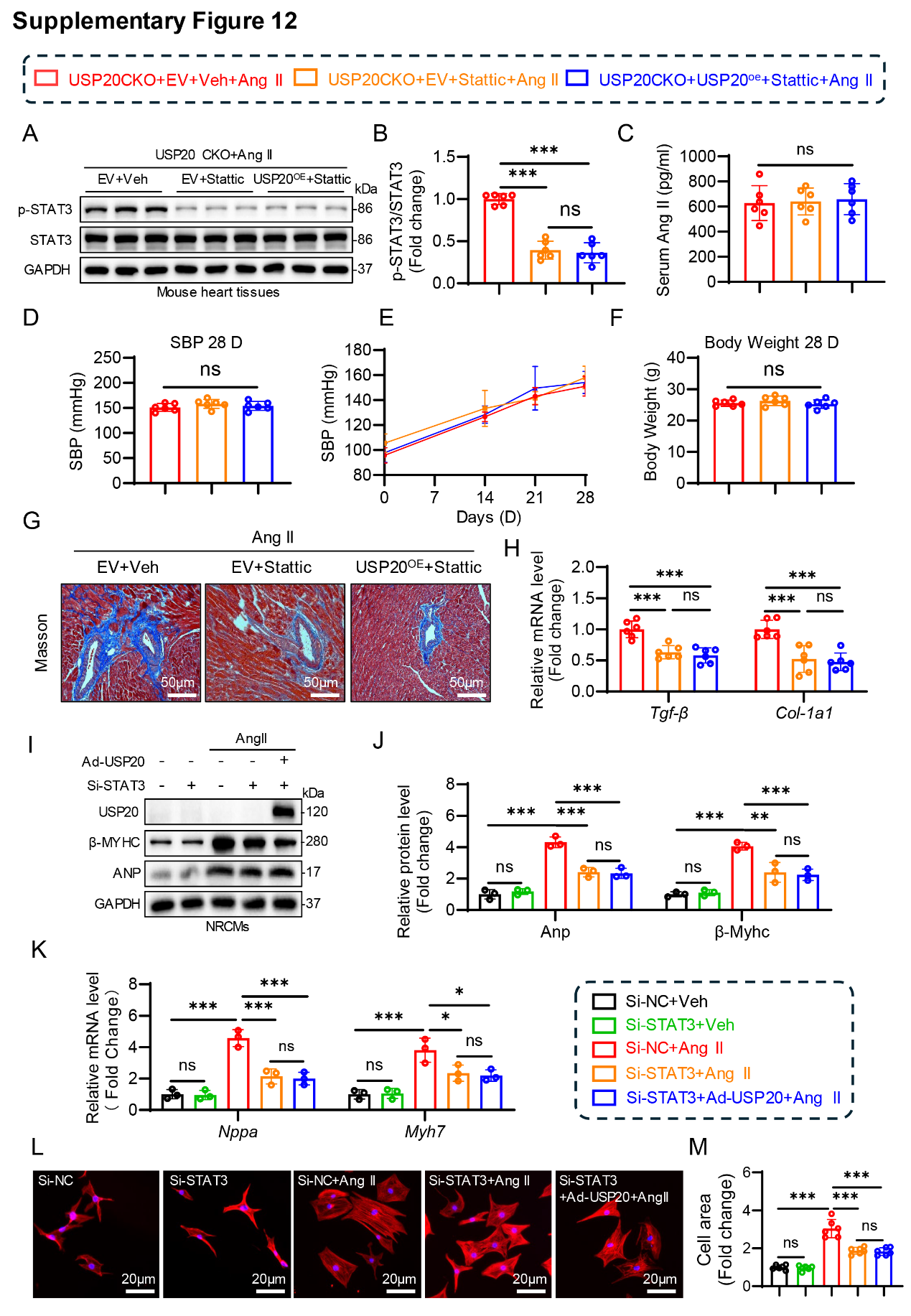
**

**Figure S12.** USP20 CKO mice were implanted with Ang II-infused osmotic mini-pump for 4 weeks. During this period, STAT3 inhibitor stattic was administered every day (10mg/kg, qd). A-B) Representative western blotting of p-STAT3 and STAT3 (A), and the densitometric quantification (B) of p-STAT3/STAT3. ***p < 0.001. ns., no significance. C) Serum Ang II level was detected by Ang II ELISA kit. D-E) Mice systolic blood pressure (SBP) during study (D) and at day 28 (E)*.* (*n* = 6). ns., no significance. F) Body weight (BW) of mice at day 28. (*n* = 6). ns., no significance. G) Representative masson stained images of perivascular regions in the heart sections. Scale bar, 50 μm. H) RT-qPCR analysis of *Tgf-β* and *Col-1a1*in heart tissues. (*n* = 6). ***p < 0.001. ns., no significance. I-M) NRCMs were infected with plasmids of Ad-Null or Ad-USP20 at MOI of 50 for 12 h, and then transfected with si-NC or si-STAT3 for 24 h, following stimulation with Ang II at 1 μM for 24 h. I-J) Representative western blotting (I) and densitometric quantification (J) of USP20, β-Myhc and Anp. (*n* = 3). **p < 0.01, ***p < 0.001. ns., no significance. K) RT-qPCR analysis of *Nppa* and *Myh7*. (*n* = 3). *p <0.05, **p < 0.01, ***p < 0.001*.* L-M) The TRITC-labeled rhodamine phalloidin staining (L) and quantitative analysis (M)*.* (*n* = 6). ***p <0.001. ns., no significance.

**Supplementary Tables**

**Supplementary Table S1**: The relative expression of marker genes in the main cell types for cell populations.

| **Cell populations** | **Marker genes** |
| --- | --- |
| Cardiomyocytes | Tnnt2, Actn2, Myh6, Myh7, Tnni3, Myl2, Myl3 |
| Fibroblasts | Mfap5, Bgn, Mgp, Dcn, Gsn, Col1a1, Col1a2,Pdgfra |
| Endothelial cells | Gpihbp1, Ly6c1, Cd36, Fabp4, Pecam1, Vwf, Eng |
| Neutrophils | Ifitm1, S100a9 |
| Macrophages | Apoe, C1qb, C1qa, Adgre1, Csf1r, Cd163, Mrc1 |
| T/NK cells | Cd3g, Nkg7, Ccl5, Il7r, Cd3d |
| Smooth muscle cells  (SMCs) | Mustn1, Myl9, Acta2, Tagln, Myh11 |
| Lymphatic endothelial cells (Lym Ecs) | Reln, Prox1, Ccl21a, Mmrn1 |
| B cells | Ly6d, Ms4a1, Ighm, Cd79a, Igkc |

**Supplementary Table S2**: The clinical characteristics of the patients.

| **Group** | **No.** | **Age** | **Sex** |
| --- | --- | --- | --- |
| Control samples  (Normal donors without heart failure) | 1  2  3 | 50  38  54 | Male  Male  Male |
| Heart failure | 1  2  3 | 65  42  61 | Male  Male  Male |

**Supplementary Table S3:** Sequences of primers for real-time qPCR assay used in study.

| **Gene** | **Species** | **Sequence (Forward)** | **Sequence (Reverse)** |
| --- | --- | --- | --- |
| Usp20 | Mouse | GCCTTCATCGTGGAGTACATCAGAC | CAGCAGCAAAGAAAGCAGCAAGG |
| Anp | Mouse | AAGAACCTGCTAGACCACCTGGAG | TGCTTCCTCAGTCTGCTCACTCAG |
| Myh7 | Mouse | CAGAACACCAGCCTCATCAACCAG | CACAGCTTCTCTTTGATGTCAC |
| Col-1a1 | Mouse | TGGCCTTGGAGGAAACTTTG | CACAGCTTCTCTTTGATGTCAC |
| Tgf-β | Mouse | CCAGATCCTGTCCAAACTAAGG | CACAGCTTCTCTTTGATGTCAC |
| Stk3 | Mouse | CCAAACGGCATGAAGAGCAGCA | CTCCTTCACTCATCGTGCTGGT |
| Pten | Mouse | TTTGAAGACCATAACCCACCACAG | TTACACCAGTCCGTCCCTTTCC |
| Eno1 | Mouse | TACCGCCACATTGCTGACTTGG | GCTTGTTGCCAGCATGAGAACC |
| Atf4 | Mouse | AACCTCATGGGTTCTCCAGCGA | CTCCAACATCCAATCTGTCCCG |
| Alkbh5 | Mouse | TCGGAACCTGTGCTTTCTCTGC | CTTCCTGAGAATGATGACCGCC |
| Carm1 | Mouse | ACAGGCTCCAAGTCCAGTAACC | GCTGCTGAGATTATAGGTGCTTCC |
| Pcna | Mouse | CAAGTGGAGAGCTTGGCAATGG | GCAAACGTTAGGTGAACAGGCTC |
| β-actin | Mouse | CCGTGAAAAGATGACCCAGA | TACGACCAGAGGCATACAG |
| Usp20 | Rat | GCAAGTGGATGAGGACGCTGATG | GTGGACTTCATTGTCTGGCTCTGG |
| Anp | Rat | GGGCTCCTTCTCCATCACC | CTCCAATCCTGTCAATCCTACC |
| Myh7 | Rat | GAGGAGAGGGCGGACATT | ACTCTTCATTCAGGCCCTTG |
| β-actin | Rat | CCTAGACTTCGAGCAAGAGA | GGAAGGAAGGCTGGAAGA |
| Usp20 | Human | TTCTACAGGAAGAGCAGCGAGGAG | GCGAAGGTGTTGAACTTGTTGAGC |
| β-actin  Carm1 promoter | Human  Mouse | CCTGGCACCCAGCACAAT  CAAGCATTGCCTCCTGAAACG | GCCGATCCACACGGAGTACT  GAGGCGGTGCTTAGCCTTC |
|  |  |  |  |

**Supplementary Table S4:** Biometric and echocardiographic parameters in Ang II-challenged mouse experiment.

| **Model 1 (Ang II model)** | **Veh** | | **Ang II** | |
| --- | --- | --- | --- | --- |
| **Parameter** | **USP20fl/fl** | **USP20 CKO** | **USP20fl/fl** | **USP20 CKO** |
|  | **n=6** | **n=6** | **n=6** | **n=6** |
| Heart rate,(bpm) | 492±55 | 479±48^ns^ | 472±38^ns^ | 474±38^NS^ |
| Diameter;s, (mm) | 1.85±0.29 | 2.6±0.32^**^ | 2.86±0.29^**^ | 3.91±0.39^###^ |
| Diameter;d, (mm) | 2.94±0.28 | 3.47±0.36^ns^ | 3.80±0.42^**^ | 4.61±0.35^##^ |
| LVAW;s, (mm) | 1.12±0.11 | 1.15±0.04^ns^ | 1.21±0.09^ns^ | 1.30±0.05^NS^ |
| LVAW;d, (mm) | 0.74±0.08 | 0.62±0.08^ns^ | 0.77±0.08^ns^ | 0.92±0.21^NS^ |

ns and *, represents USP20 CKO + Veh group or USP20^fl/fl^ + Ang II group compared with USP20^fl/fl^ + Veh group. ns, no significance. **p＜0.01. NS and #, represents USP20 CKO + Ang II group compared with USP20^fl/fl^ + Ang II group. NS, no significance. ## p＜0.01, ###, p＜0.001.

**Supplementary Table S5:** Biometric and echocardiographic parameters in TAC operation mice

| **Model 2 (TAC model)** | **Sham** | | **TAC** | |
| --- | --- | --- | --- | --- |
| **Parameter** | **USP20^fl/fl^** | **USP20 CKO** | **USP20^fl/fl^** | **USP20 CKO** |
|  | **n=6** | **n=6** | **n=6** | **n=6** |
| Heart rate,(bpm) | 499±40 | 515±39^ns^ | 552±20^ns^ | 519±70^NS^ |
| Diameter;s, (mm) | 2.23±0.19 | 2.44±0.28^ns^ | 2.91±0.19^**^ | 3.06±0.48^NS^ |
| Diameter;d, (mm) | 3.40±0.22 | 3.60±0.26^ns^ | 3.87±0.20^*^ | 4.00±0.32^NS^ |
| LVAW;s, (mm) | 1.42±0.16 | 1.14±0.12^ns^ | 1.23±0.18^ns^ | 1.36±0.13^NS^ |
| LVAW;d, (mm) | 0.70±0.10 | 0.74±0.19^ns^ | 0.84±0.12^ns^ | 0.93±0.18^NS^ |

ns and *, represents USP20 CKO + Sham group or USP20^fl/fl^ + TAC group compared with USP20^fl/fl^ + Sham group. ns, no significance., *p＜0.05, **, p＜0.01. NS and #, represents USP20 CKO + TAC group compared with USP20^fl/fl^ + TAC group. NS, no significance.

**Supplementary Table S6:** Biometric and echocardiographic parameters in Ang II infusion mice with AAV9.

| **Model 3 (AAV)** | **Veh** | **Ang II** | |
| --- | --- | --- | --- |
| **Parameter** | **WT**  **EV**  **n=6** | **WT**  **EV**  **n=6** | **WT**  **USP20^OE^**  **n=6** |
| Heart rate,(bpm) | 512±39 | 513±40^ns^ | 509±28^NS^ |
| Diameter;s, (mm) | 2.08±0.11 | 2.52±0.34^*^ | 2.30±0.28^NS^ |
| Diameter;d, (mm) | 3.20±0.22 | 3.76±0.24^**^ | 3.32±0.33^#^ |
| LVAW;s, (mm) | 1.18±0.12 | 1.33±0.13^ns^ | 1.28±0.13^NS^ |
| LVAW;d, (mm) | 0.75±0.10 | 0.94±0.10^*^ | 0.89±0.12^NS^ |

ns and *, represents WT+EV+Ang II compared with WT+EV+Veh. ns, no significance., *p＜0.05, **p＜0.01. NS and #, represents WT+USP20^OE^+Ang II compared with WT+EV+Ang II. NS, no significance., #p＜0.05.

**Supplementary Table S7:** Biometric and echocardiographic parameters in Ang II infusion USP20 CKO mice with stattic and AAV9

| **Model 4 (Stattic)** | **Ang II** | | |
| --- | --- | --- | --- |
| **Parameter** | **USP20 CKO**  **Veh+EV**  **n=6** | **USP20 CKO**  **Stattic+EV**  **n=6** | **USP20 CKO**  **Stattic+USP20^OE^**  **n=6** |
| Heart rate,(bpm) | 424±52 | 410±68^ns^ | 401±56^NS^ |
| Diameter;s, (mm) | 3.40±0.49 | 2.79±0.65^ns^ | 2.76±0.41^NS^ |
| Diameter;d, (mm) | 4.12±0.62 | 3.72±0.70^ns^ | 3.73±0.23^NS^ |
| LVAW;s, (mm) | 1.39±0.16 | 1.19±0.15^ns^ | 1.18±0.16^NS^ |
| LVAW;d, (mm) | 0.99±0.14 | 0.80±0.08^*^ | 0.82±0.05^NS^ |

ns and *, represents USP20 CKO+Stattic+EV+Ang II compared with USP20 CKO+Veh+EV+Ang II. ns, no significance., *p＜0.05; NS and #, represents USP20 CKO+Stattic+USP20^OE^+Ang II compared with USP20 CKO+Stattic+EV+Ang II. NS, no significance.

**Supplementary Table S8. Antibody information.**

| **Antibodies** | **Manufacturer** | **Cat. No.** | **Source** | **Application(s) and Dilution** |
| --- | --- | --- | --- | --- |
| USP20 | Abcam | ab72225 | rabbit | WB (1:1000)  Co-IP (2 µg per 500 µg of total protein),  IF (1:100) |
| UB | Abcam | ab134953 | rabbit | WB (1:1000) |
| UB-K63 | Abcam | ab-179434 | mouse | WB (1:1000), |
| β-MYHC | Abcam | ab172967 | rabbit | WB (1:1000), |
| Lamin B | Abcam | ab133741 | rabbit | WB (1:1000) |
| α-actinin | Abcam | ab68167 | mouse | IF(1:200) |
| Vimentin | Abcam | ab8978 | mouse | IF(1:200) |
| CD68 | Abcam | ab283654 | mouse | IF(1:200) |
| STAT3 | Cell Signaling Technology | 12640S | rabbit | WB (1:1000)  Co-IP (2 µg per 500 µg of total protein),  IF (1:100)  Cut&Tag (1:200) |
| Tyr705-STAT3 | Cell Signaling Technology | 9145S | rabbit | WB (1:1000) |
| GAPDH | Cell Signaling Technology | 5174 | rabbit | WB (1:1000), |
| ANP | Proteintech | 27426-1-AP | rabbit | WB (1:1000), |
| His | Proteintech | 6005-1-Ig | rabbit | Co-IP (2 µg per 500 µg of total protein) |
| Flag | Proteintech | 20543-1-AP | rabbit | WB (1:1000)  Co-IP (2 µg per 500 µg of total protein) |
| HA | Proteintech | 51064-2-AP | rabbit | WB (1:1000)  Co-IP (2 µg per 500 µg of total protein) |
| TNNT2 | Proteintech | 15513-1-AP | rabbit | WB (1:1000) |

Supplemental Methods and Materials

Animals

The experimental protocols involving mice were approved by the Experimental Animal Ethics Committee at the Second Affiliated Hospital of Zhejiang University college of Medicine. (Approval No. AIRB-2024-1663). GemPharmatech Co., Ltd (Nanjing, China) generated the cardiomyocyte-specific USP20 knockout mice (USP20^flox/flox^-Myh6-Cre, USP20 CKO). The cardiomyocyte-specific USP20 knockout mice (USP20^flox/flox^-Myh6-Cre, USP20 CKO) were generated using CRISPR/Cas9 technology by GemPharmatech Co., Ltd (Nanjing, China). The USP20 CKO were generated by using CRISPR/Cas9 technology. sgRNA was transcribed in vitro, donor vector was constructed Cas9, sgRNA，and donor were microinjected into the fertilized eggs of C57BL/6JGpt mice. Fertilized eggs were transplanted to obtain positive F0 mice which were confirmed by PCR and sequencing. A stable F1 generation mouse model was obtained by mating positive F0 generation mice with C57BL/6JGpt mice. The flox mice was knocked out after mating with mice expressing Cre recombinase, resulting in the loss of function of the Usp20 in cardiomyocyte. All experiments involving live animals adhered strictly to the Guide for the Care and Use of Laboratory Animals (U.S. National Institutes of Health, NIH publication no. 85-23, revised 1996). These procedures were conducted under the approval of the Institutional Animal Care and Use Committee (IACUC) of the Second Affiliated Hospital, Zhejiang University School of Medicine (Approval No. AIRB-2024-1663).

Animal experiments

A minimum of 6 animals per group was required for the study. All animal experiments were carried out and analyzed in a blinded manner. Data collection and analysis were performed by two observers unaware of the group assignment or treatment of the animals. We exclusively chose male mice for all experiments in this study. This decision was based on research indicating that estrogen and low testosterone levels might offer protection against cardiac fibrosis.

Human heart samples

Left ventricular (LV) tissues were taken from patients with terminal-stage heart failure indicated for heart transplantation. In brief, the patient’s heart was removed at the time of transplantation, and LV tissue was subsequently dissected and snap-frozen. We used LV samples from healthy hearts that were not implanted to serve as controls. All experimental protocols involving patients were approved by the Ethics Committee of the Second Affiliated Hospital Zhejiang University School of Medicine (IRB-2024-1270). The clinical characteristics of the patients are summarized in Supplementary Table S2.

Reagents

Ang II (HY-13948) and Stattic (HY-13818) were purchased from MedChemExpress (New Jersey, USA). siRNA (si-NC, si-USP20, si-STAT3) were purchased from Ribobio (Guangzhou, China). Plasmids (Flag-USP20, His-STAT3, HA-Ub, HA-K48, HA-K63) and AAV9 (USP20oe and Empty Vector) were obtained from Genechem (Shanghai, China). Antibodies against USP20 (ab72225, 1:1000), UB (ab134953, 1:1000), UB-K63 (ab179434, 1:1000), β-MYHC (ab172967, 1:1000), Lamin B (ab133741, 1:1000), α-actinin (ab68167), vimentin (ab8978) and CD68 (ab283654) were obtained from Abcam (Cambridge, UK). Antibodies against STAT3 (12640S, 1:1000), phospho (Tyr705)-STAT3 (9145S, 1:1000) and GAPDH (5174, 1:1000) were purchased from Cell Signaling Technology (MA, USA). Rabbit IgG (B900610) and antibodies against ANP (27426-1-AP, 1:1000), His (6005-1-lg, 1:1000), Flag (20543-1-AP, 1:1000), HA (51064-2-AP, 1:1000) and TNNT2 (15513-1-AP, 1:1000) were obtained from Proteintech (Hubei, China). The Rhodamine-conjugated Phalloidin (Phalloidin-Rho; cat# CA1610-300T), Masson's Trichrome Stain Kit (G1340) and Hematoxylin-Eosin staining (HE) assay kit (G1120) were purchased from Solarbio Life Sciences (Beijing, China). Stattic (19983-44-9), a STAT3 inhibitor, was purchased from MedChemExpress (New Jersey, USA).

Cardiac hypertrophy induced by Angiotensin (Ang II)

The Ang II-induced cardiac hypertrophy model was constructed by subcutaneously implanting an osmotic pump (Alzet MODEL 1004, USA) into the dorsal region of each mouse as described in our previous study.1 The 6-8-week-old mice were utilized for inducing cardiac hypertrophy by administering Ang II (1 μg/kg/min) via the osmotic pump for a duration of 4 weeks. The control group comprising an equivalent number of littermate mice received saline injections. Daily observations of the incisions on the mice were made during the first week after surgery, while blood pressure and body weight measurements were taken every 3 days. The model was considered successfully established if systolic blood pressure exceeded 150 mmHg and remained at that level for 4 weeks. Cardiomyocyte-specific overexpression of UPS20 (USP20^OE^) was achieved using recombinant adeno-associated virus serotype 9 (AAV9). AAV9s carrying Usp20 cDNA (NM-028846-3Flag) with a cardiac-specific promoter cTnT (cTNTp-MCS-SV40 PolyA, GV618, Genechem Co., Ltd, Shanghai, China) were administered via tail vein injection (2 x 10^11^ v.g.). The control group received an equivalent volume of AAV9-empty vector (EV, 2 x 10^11^ v.g.). Four weeks later, cardiac hypertrophy was induced by Ang II for 4 weeks. STAT3 inhibitor stattic (10mg/kg) was administered via oral gavage every two days during Ang II-model time periods.

Cardiac hypertrophy induced by transverse aortic constriction

The 6-8-week-old mice were administered isoflurane anesthesia via an air anesthesia machine before undergoing transverse aortic constriction (TAC).^[1]^ After appropriate skin preparation, the mice were positioned supine and intubated using an endotracheal tube connected to mechanical ventilation support. Make a sternal incision at its proximal region, allowing access into the thoracic cavity through placement of a thoracotomy device; Constriction of the aortic arch occurred by ligation between the left common carotid artery and innominate artery utilizing sterile 6-0 nylon suture encircling around a blunt 27-gauge needle which was subsequently withdrawn. Sham-operated mice underwent the same procedure without aortic ligation. Mice were subjected TAC or sham for 4 weeks.

Adeno-associated virus and viral delivery protocol

Cardiomyocyte-specific overexpression of UPS20 (USP20^OE^) was achieved using recombinant adeno-associated virus serotype 9 (AAV9). AAV9s carrying Usp20 cDNA (NM_028846-3Flag) with a cardiac-specific promoter cTnT (cTNTp-MCS-SV40 PolyA, GV618, Genechem Co., Ltd, Shanghai, China) were administered via tail vein injection (2 x 10^11^ v.g.). The control group received an equivalent volume of AAV9-empty vector (EV, 2 x 10^11^ v.g.). After the injections (EV or USP20^OE^) were performed for 4 weeks, the Ang II-model was established. Mice were harvested 4 weeks after Ang II. An experimental subgroup included the STAT3 inhibitor stattic-treated mice. Stattic (10mg/kg) was administered via oral gavage every two days during Ang II-model timeframe.

Transthoracic echocardiography

Measurement of cardiac function by echocardiography was performed as described previously.^[2]^ Echocardiographic measurements were performed on mice using a Visual Sonics Vevo® 2100 Imaging System (Visual Sonics, Toronto, Canada) with a 40 MHz MicroScan transducer (model MS-550D)18,27,34. Mice were anesthetized with isoflurane (2.5% isoflurane for induction and 0.5% for maintenance). Heart rate and left ventricular (LV) dimensions were measured from 2-D short-axis under M-mode tracings at the level of the papillary muscle. LV mass and functional parameters such as percentage of ejection fractions (EF%) and fractional shortening (FS%) were calculated using the above primary measurements and accompanying software.

Histological staining

Paraffin-embedded myocardium sections were stained with hematoxylin and eosin (HE, G1120, Solarbio, Beijing, China). Fibrosis was assessed using Masson's trichome staining (S8060, Solarbio, Beijing, China). Cardiomyocyte area was measured with wheat germ agglutinin (WGA-FITC, GTX01502, GeneTex, Texas, USA) on optimal cutting temperature (OCT)-embedded sections following the manufacturer’s instructions. The images were captured under a microscope (Leica, Germany) at a magnification of 20× and quantified by NIH ImageJ software (Java 1.8.0 USA). OCT sections were incubated with anti-USP20 (Abcam), anti-α-actinin (Abcam), anti-CD68 (Abcam), or anti-vimentin (Abcam), followed by secondary antibodies: anti-mouse IgG Alexa Fluor 488 (HuaBio) or anti-rabbit IgG Alexa Fluor 594 (HuaBio). Nuclei were stained with DAPI (Solarbio, Beijing, China). Images were captured using an optical or fluorescence microscope (Leica, Germany). Three to six sections per group were analyzed, with six random microscopic fields.

Enzyme linked immunosorbent assay (ELISA)

The experimental procedures were conducted in compliance with the manufacturer's instructions for the Elisa kit. The immune signals of Ang II (E-EL-H5518c, Elabscience, China) in serum of mice were assessed through a series of steps including coating, blocking, sample addition, primary antibody incubation, washing, secondary antibody incubation, further washing, coloration, termination, and other necessary procedures.

Cell and cell culture

The mouse cardiomyocytes-HL-1, NIH3T3 cells and HEK293T cells were obtained from the Shanghai Institute of Biochemistry and Cell Biology (Shanghai, China). All cells were cultured in the DMEM medium containing 10% fetal bovine serum (FBS) and 1% penicillin/streptomycin. The cultures were maintained in a temperature-controlled incubator at 37 °C with an atmosphere consisting of 21% O_2_ and 5% CO_2_.

Isolation and culture of neonatal rat cardiomyocytes (NRCMs)

Neonatal rat cardiomyocytes were isolated from P0 neonatal rat using the Miltenyi Neonatal Heart Dissociation Kit (Miltenyi, Germany).^[3]^ Initially, P0 neonatal rat were submerged in 75% ethanol for 10-15 seconds to prevent contamination. A small incision was made at the left sternum, and the heart was gently extracted and placed in room temperature PBS buffer to allow it to pump out excess blood. Blood clots and surrounding tissues were carefully removed with tweezers. Meanwhile, Enzyme mix 1 (62.5 μl Enzyme P and 2300 μl buffer X) was preheated in a 37°C incubator for 5 minutes. After preheating, Enzyme Mix 1 was combined with Enzyme Mix 2 (12.5 μl Enzyme A, 100 μl Enzyme D, and Buffer Y). The heart was placed in this mixture and incubated at 37°C for 15 minutes. The mixture was then removed, and the heart was gently pipetted 20 times using a sterile 5 ml pipette. After a final 15-minute digestion, the heart tissues turned into a cloudy solution after being pipetted with a 1 ml pipette. The enzyme solution was neutralized with high-glucose DMEM containing 10% Gibco FBS, and the mixture was filtered through a 70 μm cell strainer to remove undigested tissues. The filtrate was centrifuged at 1000g for 5 minutes, and the supernatant was discarded, leaving a cell pellet containing all heart cells. To purify the cardiomyocytes, the cells were resuspended in fresh high-glucose DMEM with 10% Gibco FBS and plated in a 10 cm dish. Differential adhesion was allowed for 20 minutes in a 37°C CO_2_ incubator, during which time cardiomyocytes remained in suspension while fibroblasts adhered to the dish. The medium containing the cardiomyocytes was gently collected for further experiments.

Cell transfection

USP20 and STAT3 were silenced in NRCMs using small interfering RNA (siRNA) with the following target sequences: GGAAGACCTAGCCAAACTT for Usp20, and GCAGGTCTAAACAGAAA for Stat3 (RIBOBIO, Guangzhou, China). Transfections were carried out using LipofectamineTM RNAiMAX (Thermo Fisher Scientific, Germany). Plasmids including Flag-USP20 (Mouse), Flag-USP20-C154A (Mouse), Flag-USP20-H645A (Mouse), Flag-USP20-del(145-687aa)(Mouse), Flag-USP20-del(689-782aa)(Mouse), Flag-USP20-del(791-894aa)(Mouse), His-STAT3-WT (Mouse and Human), His-STAT3-K87R (Mouse), His-STAT3-K97R (Mouse), His-STAT3-K161R (Mouse), His-STAT3-K177R (Mouse), His-STAT3-K244R (Mouse), His-STAT3-K294R (Mouse), His-STAT3-K348R (Mouse), His-STAT3-K354R (Mouse), His-STAT3-K548R (Mouse), His-STAT3-K551R (Mouse), His-STAT3-K601R (Mouse), His-STAT3-K615R (Mouse), His-STAT3-K626R (Mouse), His-STAT3-K631R (Mouse), His-STAT3-K707R (Mouse), His-STAT3-Mut1 (del-NTD) (Human), His-STAT3-Mut1 (del-CCD) (Human), His-STAT3-Mut1 (del-DBD)(Human), His-STAT3-Mut1 (del-LD) (Human), His-STAT3-Mut1 (del-SH2)(Human), His-STAT3-Mut1 (del-TAD) (Human), HA-Ub (Mouse), HA-K48 (Mouse), and HA-K63 (Mouse) were sourced from Genechem (Shanghai, China) and expressed using LipofectAMINE™ 3000 (Thermo Fisher Scientific, Germany). For NRCMs, USP20 was overexpressed by infecting the cells with adenovirus particles carrying USP20 (Ad-USP20) or USP20-H645A (Ad-USP20-H645A) at a multiplicity of infection at MOI of 50 for 12 h. Adenovirus with no insert (Ad-null) was used as the control. The adenoviral vectors were sourced from Genechem, Shanghai, China.

TRITC-phalloidin fluorescent staining

The cell culture medium was removed, and the cells were washed once with PBS. The cells were then fixed with 4% paraformaldehyde for 15 minutes at room temperature, followed by 2-3 additional PBS washes. To enhance permeability, the cells were treated with PBS containing 0.1% Triton X-100 for 3-5 minutes, then washed again 2-3 times with PBS. TRITC-Phalloidin conjugated solution (CA1610, Solarbio, China) was added and incubated at room temperature for 30 minutes. Afterward, the excess dye was removed by rinsing the cells 2-3 times with PBS, and the nuclei were stained with antifade mounting medium containing DAPI (S2110, Solarbio, China). The staining results were captured using a fluorescence microscope for statistical analysis.

Confocal microscopy

NRCMs were incubated overnight at 4°C with anti-USP20 (1:200, ab72225, Abcam) and anti-STAT3 (1:200, 12640S, CST). NIH3T3 cells were transfected with expression plasmids were similarly incubated with anti-Flag (1:200, 20543-1-AP, Proteintech) and anti-His (1:200, 6005-1-lg, Proteintech) overnight at 4°C. Both cell types were then incubated with TRITC-labeled secondary antibody (1:1000, Abcam) or Alexa Fluor488-labeled secondary antibody (1:1000, Abcam) for 1 h at room temperature. DAPI was used for nuclear staining, and images were obtained with a laser confocal microscopy system (LSM 980 with Airyscan 2, Zeiss, German) with dual line switching excitation (488 nm and 543 nm) and emission (500 - 560 nm for FITC, 580 - 670 nm for Alexa Fluor® 594) filter sets.

RNA-sequencing (RNA-Seq) and single-cell RNA sequencing (scRNA-Seq) analysis

RNA-Seq analysis was conducted on heart tissues from USP20^fl/fl^ and USP20 CKO mice treated with Ang II infusion or Ang II-treated and vehicle-treated mice. Total RNA was extracted using TRIzol reagent (Invitrogen, USA). Hearts from Ang II-treated and vehicle-treated mice were dissociated into single cells using a dissociation solution. These single-cell suspensions were then loaded onto the 10X chromium platform to capture individual cells using the 10X genomics Chromium Single-Cell 3’ kit.^[4,5]^ The cDNA amplification, library construction, sequencing and further analysis of RNA-Seq and scRNA-Seq were entrusted to LC bio-technology Co., Ltd. (Hangzhou, China).

Liquid chromatography-mass spectrometry/mass spectrometry (LC-MS/MS) analysis

USP20 antibody was added to a sample of Ang II-stimulated heart tissue lysate, with IgG serving as a negative control. LC-MS/MS analysis was then performed by PTM Bio Co., Ltd (Hangzhou, Zhejiang, China).^[6]^ Based on the score and mass of the detected proteins, we identified substrate proteins that could bind to USP20. Target proteins were selected based on the following criteria: 1. Protein signal ratio (USP20-specific antibody experimental group/IgG control group) > 5-fold; 2. Exclusive identification by the experimental group to identify USP20 substrate proteins. The target proteins obtained above underwent further screening with high confidence using unique peptides > 5 and Sequence coverage > 10% as conditions.

Ubiquitylome analysis

HL-1 cells were transfected with either EV or Flag-USP20 plasmids. Proteins were extracted and digested twice with trypsin to obtain peptides. These peptides were enriched for ubiquitinated peptides by dissolving them in IAP buffer and incubating them with anti-K-Ubiquitin antibody beads (5562, CST) at 4°C overnight. LC-MS/MS and ubiquitylome analysis were conducted by PTM Bio Co., Ltd (Hangzhou, Zhejiang, China).^[7]^

Cleavage under targets and tagmentation (CUT&Tag) assay

HL-1 cells were transfected with USP20 or empty vector (EV) plasmids and then stimulated with Ang II at 1 μM for 24 h. The cells were harvested for the CUT&Tag assay (NovoNGS CUT&Tag 3.0 high-sensitivity kit, N259-YH01, Novoprotein, China): Harvested cells were resuspended in 90 μL wash buffer, and mixed with 10 μL ConA beads, incubating for 10 minutes at RT. The cells were then incubated with anti-STAT3 for 2 h at RT, followed by incubation with the secondary antibody for 1 h at RT. Transposome pA-Tn5 was then added and incubated with the cells for 1 h at RT. DNA fragments were isolated using Tagment DNA extract beads and dissolved in 37 μL TE buffer. The DNA was then amplified using N5 and N7 primers and subjected to high-throughput sequencing (PTM Bio Co., Ltd, Hangzhou, Zhejiang, China) and qPCR. Primer sequences for CUT&Tag are provided in Table S3.

Luciferase reporter assay

NIH3T3 cells were seeded in 24-well plates one day prior to transfection (1×10^5^ cells/well). The Renilla plasmid (pRL-TK) was co-transfected with the specified vectors, and firefly luciferase activity from each sample was normalized to the corresponding Renilla luciferase activity. The PPRE-Luc vector was transfected alongside NC-STAT3 or Ad-STAT3. Cells were subsequently harvested, and luciferase activity was quantified. To determine the STAT3 binding region on the Carm1 promoter, the genomic fragment from -1659 to -1650 (TGCCGGGAAG) of the Carm1 promoter was amplified and cloned into the pGL4.16 vector. A sequence consisting of AGACTGTATG (-1659 to -1650) was cloned into pGL4.16 as a negative control. The constructed pGL vector was transfected into NIH3T3 cells with or without the STAT3 expression plasmid, and luciferase activity was then measured and analyzed as described previously.^[8,9]^ The promotor region of mouse Carm1 including transcription factor STAT3 binding site (TGCCGGGAAG) was amplified from genomic DNA of mouse NIH3T3 cells. The reported construct was generated by linking the Carm1 promotor (WT or Mut) to PGL3-basic vector. NIH3T3 was transfected with mouse STAT3 overexpression plasmid in 96-well plate, next infected with the indicated luciferase constructs and internal control plasmid phRL-TK. The firefly luciferase and Renilla luciferase activity was measured according to the manual of Dual-luciferase reporter assay kit (Promega cat #E2920).

Western blot

Cells or tissues were sufficiently lysed by the RIPA lysis buffer (P0013B, Beyotime, China) containing protease and phosphatase inhibitor cocktail (78442, Thermo Fisher, USA), to obtain the total protein in the samples. And nuclear proteins were extracted with their extraction kits (P0028, Beyotime, Shanghai, China). After quantified by BCA protein assay kit (23225; Thermo Fisher, USA), the protein extracts were concentrated and separated by SDS-PAGE electrophoresis and transferred to polyvinylidene difluoride (PVDF) membranes (ISEQ00010, Merck, USA). Next, these PVDF membranes were blocked with 5% skim milk for 1 h at room temperature, incubated with primary antibody overnight at 4 ° C, washed with TBST, and ultimately incubated with HRP (horse radish peroxidase)-conjugated secondary antibody for 1 h at room temperature. The visualization of target proteins was achieved by employing electrochemiluminescence (ECL) reagents (Merck, USA) in conjunction with a western blot detection system (Amersham ImageQuant 800, UK). The Image Lab 6.0.1 software (BIO-RAD, US) was employed for quantitative analysis.

Co-immunoprecipitation (Co-IP)

The tissues (10 mg) or cells (1x10^7^) were adequately lysed at 4°C in a lysis buffer containing protease inhibitor cocktail. After centrifugation, the supernatant was collected. Each supernatant sample was treated with the addition of protein A+G agarose beads (Thermo Fisher Scientific, Germany) and shaken at 4°C for 1 h. Following centrifugation, the supernatant was retained to remove the proteins which exhibited specificity towards the beads. A portion of the lysate was obtained from the isolated supernatant, serving as input samples for later assays. The remaining samples were supplemented with corresponding primary antibodies, while the control group received normal IgG antibodies of the same species. In order to bind adequately to the primary antibody, the samples were incubated overnight at 4 °C with gentle agitation. The next day, protein A+G agarose beads were added to each sample and incubated again for 2h at 4 °C with soft shaking. After centrifugation, the supernatant was discarded and the rest of the samples was washed and mixed with loading buffer to prepare WB samples for detection. To eliminate the influence of heavy and light chains, secondary antibodies (ab157532, ab131368, Abcam) that are free of these chains will be used in Co-IP experiments.

RNA extraction and real-time quantitative polymerase chain reaction (RT-qPCR)

The RNA was extracted through the TRIzol method and conducted to quality inspection, and performed reverse transcription according to the instructions provided with the RT SuperMix (R333, Vazyme, China). A mixture of SYBR Green master mix (Q711, Vazyme, China), cDNA, and primers (purchased from Sangon Biotech Co., Ltd., Shanghai, China) was prepared and utilized for RT-qPCR analysis on a LightCycler 480 II instrument (Roche, Switzerland). The reaction conditions were implemented in accordance with the product description of SYBR Green master mix. The primer sequences utilized in this study are listed in Supplemental Table S3.

Ethical Approval

All animal protocols were approved by the Institutional Animal Care and Use Committee (IACUC) of the Second Affiliated Hospital, Zhejiang University School of Medicine (AIRB-2024-1663) and were performed in accordance with the National Institutes of Health Guide for the Care and Use of Laboratory Animals. All experimental protocols involving humans were approved by the Ethics Committee of the Second Affiliated Hospital Zhejiang University School of Medicine (IRB-2024-1270).

Funding

This study was supported by the National Natural Science Foundation of China (82170242, 82030014, 82370256, 81570454, U22A20267, U21A20338); State Key Laboratory of Transvascular Implantation Devices (012024015, 012024010); National Key R&D Program of China (2023YFA1800700).

REFERENCES

[1] J. Han, S. Dai, L. Zhong, X. Shi, X. Fan, X. Zhong, W. Lin, L. Su, S. Lin, B. Han, J. Xu, X. Hong, W. Huang, B. Ye, Hypertension. 2022, 79, 2505-2518.

[2] F. Gao, T. Liang, Y.W. Lu, X. Fu, X. Dong, L. Pu, T. Hong, Y. Zhou, Y. Zhang, N. Liu, F. Zhang, J. Liu, A.P. Malizia, H. Yu, W. Zhu, D.B. Cowan, H. Chen, X. Hu, J.D. Mably, J. Wang, D.Z. Wang, J. Chen, Nat Commun. 2023, 14, 1595.

[3] F. Gao, T. Liang, Y.W. Lu, L. Pu, X. Fu, X. Dong, T. Hong, F. Zhang, N. Liu, Y. Zhou, H. Wang, P. Liang, Y. Guo, H. Yu, W. Zhu, X. Hu, H. Chen, B. Zhou, W.T. Pu, J.D. Mably, J. Wang, D.Z. Wang, J. Chen, Circulation. 2023, 148, 1887-1906.

[4] S. Yamada, S. Nomura, Int J Mol Sci. 2020, 21.

[5] Z. Ren, P. Yu, D. Li, Z. Li, Y. Liao, Y. Wang, B. Zhou, L. Wang, Circulation. 2020, 141, 1704-1719.

[6] B. Ye, H. Zhou, Y. Chen, W. Luo, W. Lin, Y. Zhao, J. Han, X. Han, W. Huang, G. Wu, X. Wang, G. Liang, Circ Res. 2023, 132, 465-480.

[7] J.G. Abelin, E.J. Bergstrom, K.D. Rivera, H.B. Taylor, S. Klaeger, C. Xu, E.K. Verzani, C. Jackson White, H.B. Woldemichael, M. Virshup, M.E. Olive, M. Maynard, S.A. Vartany, J.D. Allen, K. Phulphagar, M. Harry Kane, S. Rachimi, D.R. Mani, M.A. Gillette, S. Satpathy, K.R. Clauser, N.D. Udeshi, S.A. Carr, Nat Commun. 2023, 14, 1851.

[8] H. Li, D. Hu, H. Fan, Y. Zhang, G.D. LeSage, Y. Caudle, C. Stuart, Z. Liu, D. Yin, J Biol Chem. 2014, 289, 23075-23085.

[9] X. Zheng, T. Zhong, F. Yu, J. Duan, Y. Tang, Y. Liu, M. Li, D. Sun, D. Yin, Front Cardiovasc Med. 2022, 9, 951463.
